# Halogen Bond‐Driven Ligand Displacement: Co‐Crystal Lattice Versus Coordination Bonds

**DOI:** 10.1002/chem.202404784

**Published:** 2025-04-17

**Authors:** Yury V. Torubaev, Omer Shaashua, Savion Braunstein, Doron Pappo

**Affiliations:** ^1^ Department of Chemistry Ben‐Gurion University of the Negev Beer‐Sheva 84105 Israel

**Keywords:** co‐crystals, coordination bond, crystallization, halogen bond, lattice energy

## Abstract

Coordination bonds are generally stronger than halogen bonds; however, the Jahn–Teller effect in d⁹ Cu(II) and the trans influence of the oxo‐ligand in vanadyl (V═O) acetylacetonates can weaken N→Cu/V bonds, bringing them closer to the upper range of halogen bond strength. The study investigates the interactions between transition metal acetylacetonate complexes, M(acac)_2_(L) (M─Cu(II), V(IV) = O; L = amine ligands), and halogen bond (XB)‐donor co‐formers, particularly 1,4‐diiodotetrafluorobenzene (1,4‐DITFB). The co‐crystallization experiments reveal an unusual ligand displacement phenomenon wherein the expected M(acac)_2_(L)·1,4‐DITFB complexes fail to form, instead yielding separate M(acac)_2_·1,4‐DITFB and L·1,4‐DITFB co‐crystals. Computational studies reveal that while XB interactions alone may be insufficient to disrupt the M─N coordination bond, they can induce ligand displacement when amplified by the lattice stabilization of the resulting halogen‐bonded co‐crystals, particularly in Jahn–Teller distorted d⁹ Cu(II) and trans‐influenced V(IV) = O complexes interacting with halogen bond donors.

## Graphical abstract

Strong halogen bond donor (1,4‐diiodotetrafluorobenzene) can induce the amine ligand displacement in Jahn–Teller affected Cu(acac)_2_(amine) and in trans influenced VO(acac)_2_(amine) complexes, resulting the formation of respective co‐crystals: M(acac)_2_·1,4‐DITFB and (amine)·1,4‐DITFB. The driving force behind this unprecedented cleavage of the metal‐nitrogen coordination bond is the halogen bond stabilized co‐crystal lattice

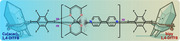

## 1 Introduction

Since Hassel's pioneering work on halogen bond (XB) assisted crystal engineering in the 1960s,^[^ ^1^, ^2^, ^3^ ^]^ the nature and relative strength of the halogen bonds compared to covalent and other “classic” non‐covalent bonds remain a central question. In structural studies, the XBs ^[^ ^4^ ^]^ are often considered in competition and interaction with concomitant non‐covalent interactions, such as hydrogen bonding, π‐stacking, and others.^[^ ^5^, ^6^, ^7^, ^8^, ^9^, ^10^, ^11^, ^12^, ^13^, ^14^ ^]^

Beyond solid‐state and material chemistry applications, where XBs are used to achieve specific packing arrangements and molecular frameworks, halogen and chalcogen bonds (ChBs) are strong enough to participate in solution‐phase processes,^[^ ^15^ ^]^ such as organocatalysis^[^ ^16^, ^17^, ^18^ ^]^ and extraction.^[^ ^19^ ^]^ From a thermodynamic perspective, XBs are now understood to range from weak (5–15 kJ/mol) to strong (40–50 kJ/mol),^[^ ^20^, ^21^ ^]^ overlapping at the lower end with van der Waals interactions (1–5 kJ/mol) ^[^ ^22^ ^]^ and at the upper end with 3‐center‐4‐electron,^[^ ^23^ ^]^ weak coordination bonds such as Ru─P in [(PH_3_) (NHC)Cl_2_Ru═CH_2_] (25 kJ/mol) ^[^ ^24^ ^]^ and hypervalent bonds^[^ ^25^, ^26^, ^27^ ^]^ This positions them as part of a continuum from covalent to van der Waals interactions rather than as standalone phenomena.^[^ ^27^, ^28^ ^]^

The metal–ligand bond energy in coordination and organometallic compounds varies widely (from 25 kJ/mol in metal‐phosphine pre‐catalysts^[^ ^29^ ^]^ to 380 kJ/mol in Cp_2_Fe^[^ ^30^ ^]^) making it challenging to generalize the strength of metal–ligand coordination bonds.^[^ ^31^ ^]^ The benchmark sets of metal–ligand bond enthalpies usually contain relatively strong ones in the range of ∼100 – 250 kJ/mol.^[^ ^32^, ^33^ ^]^

**Figure 1:**
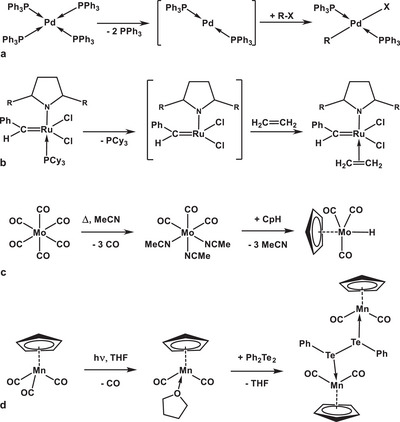
Ligands displacement as a key step in (a,b) pre‐catalyst activation ([(Ph_3_P)_4_Pd], [(PCy_3_) (NHC)Cl_2_Ru═CHPh]) and in (c,d) synthetic procedures to substitute inert CO groups in transition metal carbonyls Mo(CO)_6_ and CpMn(CO)_3_ for the labile MeCN ligands, which can be conveniently substituted for the desired ligands.

**Figure 2:**
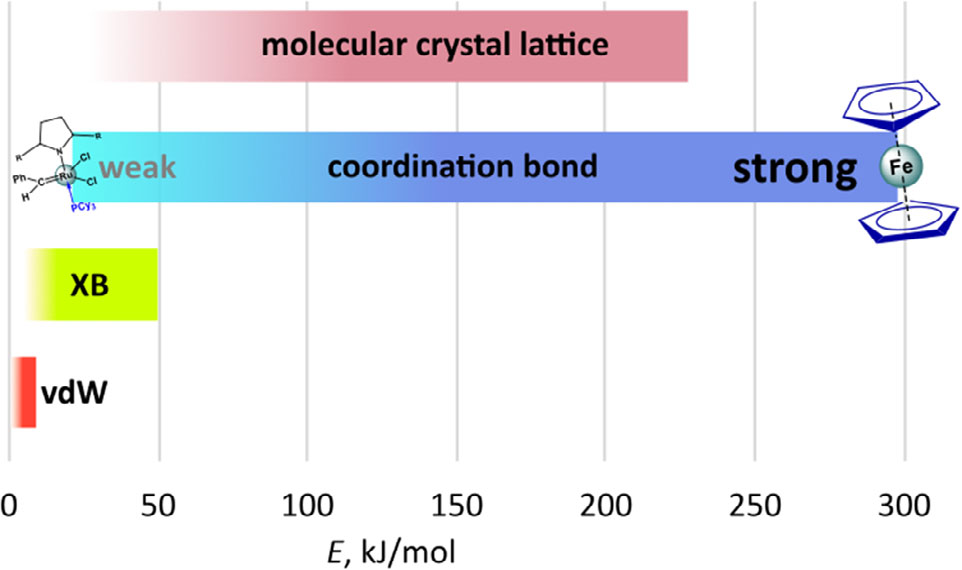
Energy scale of van der Waals bonds (vdW), halogen bonding (XB), and coordination bonds.

“Weak bonding” in this context does not carry any negative connotations, as the displacement of weakly bound ligands is a key step in transition metal catalysis^[^ ^24^, ^29^, ^34^ ^]^ and in coordination chemistry^[^ ^35^, ^36^ ^]^ (Figure 1):

In the practical context of crystal engineering, metal complexes with bond energies greater than 60 kJ/mol are considered stable, exhibiting a balance between kinetic accessibility and thermodynamic stability.^[^ ^37^ ^]^ These bonds are robust enough to provide structural integrity while being labile enough to dissociate under relatively mild conditions in solution, facilitating the formation of coordination polymers and MOF architectures.^[^ ^37^ ^]^ At the lower end of the stability range (around 60 kJ/mol), metal–ligand bond energy approaches that of strong XBs (40–50 kJ/mol, (Figure 2).

**Figure 3:**
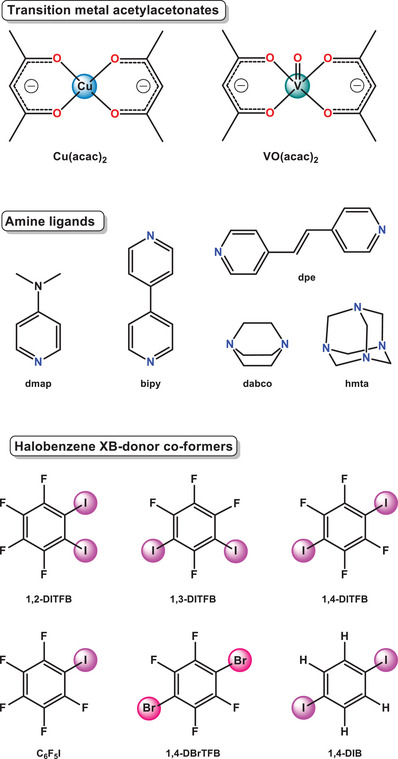
Metal complexes, amine ligands, and halo‐perfluorobenzene coformers.

This overlap raises an intriguing question: can XBs exhibit strength and reactivity comparable to those of coordination bonds, thereby actively influencing the metal complexation equilibrium in a process of crystal engineering? Earlier it was reported that competition between halogen bonding (C─X⋯Cl─Cu; X = Cl, Br, I) and weak Cu⋯Cl “semi‐coordinate” interactions plays a key role in directing polymorphism in (halo‐pyridine)dihalocopper(ii) complexes.^[^ ^38^, ^39^ ^]^ In this study of copper (II) and vanadyl acetylacetonate complexes (Figure 3), we demonstrate that XB‐assisted crystal packing can indeed compete in the “heavier weight category” of coordination bonds. Copper(II) and vanadyl(IV) acetylacetonate complexes with amine ligands and halofluorobenzene XB‐donors were chosen as the model compounds for this study (Figure 3). Acetylacetonates and other β‐diketonates of Ni(II), Co(II), and Cu(II) have already been employed as building blocks in crystal engineering to test the limits of XB‐directed co‐crystallization in coordination chemistry, demonstrating both stability and XB‐acceptor properties.^[^ ^40^, ^41^, ^42^ ^]^

The octahedral distortion in copper acetylacetonate complexes is a notable example of the Jahn–Teller effect, leading to the axial elongation of Cu‐ligand bonds.^[^ ^43^, ^44^ ^]^

In vanadyl complexes, the elongation of V─N and V─O distances in the trans position to the oxo‐ligand is also quite pronounced,^[^ ^45^, ^46^, ^47^ ^]^ and such destabilization is considered a part of the catalytic mechanism for some vanadyl complexes.^[^ ^48^ ^]^ Consequently, Cu(acac)₂(L) and VO(acac)₂(L) (L = amine ligand) were chosen as XB‐acceptor co‐crystallization partners for halobenzene XB‐donor coformers (Figure 3).

Amine ligands L in the M(acac)₂(L) series have a well‐documented history as XB‐acceptors. For instance, 4‐dimethylaminopyridine (**dmap**)^[^ ^13^ ^]^ and 1,2‐di(4‐pyridyl)ethylene (**dpe**) co‐crystals with diiodotetrafluorobenzenes (DITFBs) feature exemplary short I···N XBs, which have been the subject of experimental and theoretical studies of I···N XBs.^[^ ^13^, ^49^, ^50^, ^51^, ^52^ ^]^ Other N‐donor coformers and ligands used in this study, including 1,4‐diazabicyclo[2.2.2]octane (**dabco**), hexamethylenetetramine (**hmta**) and 4,4′‐**bipy**ridine (**bipy**) (Figure 3), have also appeared in previous XB studies.^[^ ^26^, ^53^, ^54^, ^55^, ^56^, ^57^ ^]^

## 2 Results and Discussion

### 2.1 Patterns of M(acac)_2_(amine) (M═Cu, VO) Interactions With XB‐Donor Coformers

**Figure 4:**
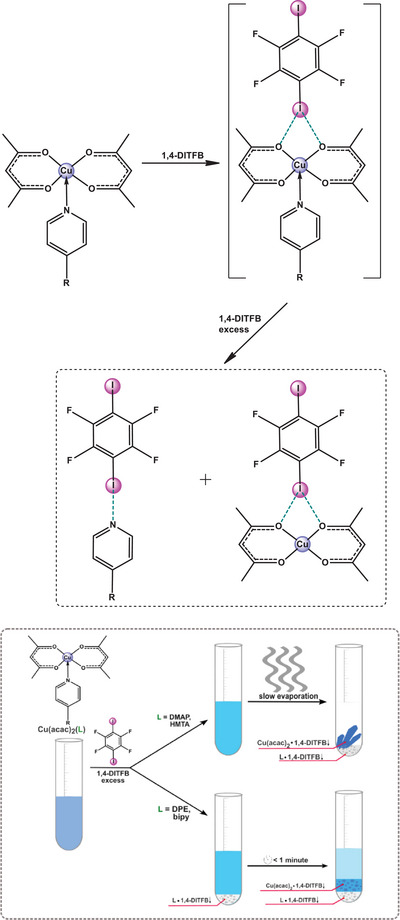
Reaction scheme for Cu(acac)_2_(L) including the assumed transient complex [Cu(acac)_2_(L)···1,4‐DITFB)]. See also Figures S1 and S2 for more illustrations.

In our ongoing investigation of transition metal complexes as building blocks in XB‐assisted crystal engineering^[^ ^58^, ^59^, ^60^, ^61^, ^62^ ^]^ we noticed that co‐crystallization of Cu(acac)_2_(**dmap**) with XB‐donor co‐former 1,4‐DITFB, instead of the expected Cu(acac)_2_(**dmap**)·1,4‐DITFB (1:1) co‐crystal, resulted in two separate co‐crystals: **dmap**·1,4‐DITFB (2:1) and Cu(acac)_2_·1,4‐DITFB (1:1) (Figure 4).

Although both resulting co‐crystals are already known,^[^ ^49^, ^50^, ^63^, ^64^ ^]^ such an uncommon ligand displacement event prompted us to investigate this phenomenon more systematically, as it is likely induced by an XB donor. We studied the ligand displacement in a series of metal acetylacetonates, M(acac)_2 _L (M = Cu(II) and vanadyl(IV) V = O), whereas L is an amine ligand 4‐dimethylaminopyridine (**dmap**), (E)‐1,2‐di(4‐pyridyl)ethylene (**dpe**), hexamethylenetetramine (**hmta**), 1,4‐diazabicyclo[2.2.2]octane (**dabco**), or 4,4′‐bipyridine (**bipy**). These complexes paired with various XB donor co‐formers: iodopentafluorobenzene (C₆F₅I), 1,2‐, 1,3‐, and 1,4‐DITFB), 1,4‐dibromotetrafluorobenzene (1,4‐DBrTFB), and 1,4‐diiodobenzene (1,4‐DIB) (Figure 3). This allowed us to probe the effects of metal center, ligand basicity, halogen bond strength, and XB‐donor co‐former symmetry on the outcome of their co‐crystallization with M(acac)_2_(L).

Visual observation and UV spectra indicate that diluted (10^−4 ^M) mixtures of Cu(acac)_2_ with amine ligands L (L = **dmap**, **bipy**, **dabco**, or **hmta**) remain stable in dichloromethane (DCM) and methanol in the presence of equimolar amount of 1,4‐DITFB. Displacement of the amine ligand from 1:2 stoichiometric solutions of Cu(acac)_2 _L and 1,4‐DITDB with the formation of co‐crystalline Cu(acac)_2_·1,4‐DITFB (1:1) and L·1,4‐DITFB (1:1), occur only when L = **dpe**. For the other amine ligands (L = **dmap**, **dpe**, **dabco**, **hmta**, and **bipy**), the modified conditions are required: (a) slow evaporation of the solvent, (b) cooling the solution at 4°C for 24 h, (c) performing the reaction in concentrated solution in DCM or MeOH, (d) addition of antisolvent (hexane) or (f) significant (3‐fold and more) excess of 1,4‐DITFB (Figuress S1, S2, S4, S11a–c).

**Figure 5:**
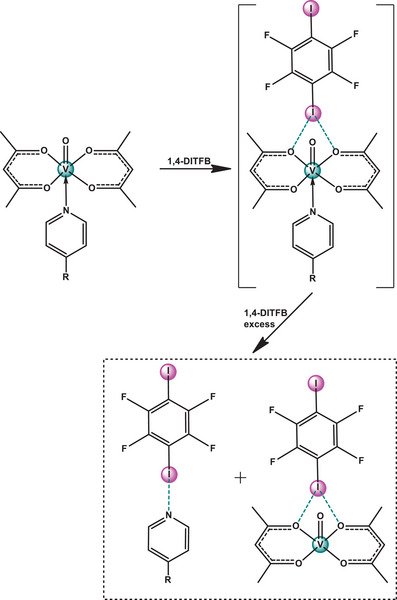
Schematic formation of L·1,4‐DITFB and VO(acac)_2_·1,4‐DITFB co‐crystals from VO(acac)_2_(L).

In the case of VO(acac)_2_(L) (L = **dmap** and **dpe**) the pattern of interaction with the stoichiometric amounts of 1,4‐DITFB is generally the same as for Cu(acac)_2_(L) (Figures 5, S3, S7). Vanadyl acetylacetonate readily affords amine derivatives in DCM solution^[^ ^65^ ^]^ which is signified by an instant color change from its inherent turquoise to the various shades of green. The addition of 2 equivalents of 1,4‐DITFB produces white precipitation only in the case of L = **dpe**, while for L = **dmap**, even the 5‐fold excess of solid 1,4‐DITFB solutions does not result in any notable color change or precipitation. Similarly to Cu(acac)_2_(L) complexes, the slow evaporation of resulting green mixtures of VO(acac)_2_(L) and 1,4‐DITFB yields yellowish‐green needles of the known VO(acac)_2_·1,4‐DITFB co‐crystals^[^ ^64^ ^]^ and colorless L·1,4‐DITFB (Figure S12).

Therefore, the major difference between Cu(acac)_2_(L) and VO(acac)_2_(L) is that VO(acac)_2_·1,4‐DITFB (1:1) co‐crystals does not precipitate from the reaction mixture as readily as Cu(acac)_2_·1,4‐DITFB do. Such a notable difference in their solubility is likely due to the different stacking patterns of square‐planar Cu(acac)_2_ and square‐pyramidal VO(acac)_2_,^[^ ^64^ ^]^ rather than the effect of I···O_acac_ XB. The latter has similar geometry and energy: the calculated intermolecular bonding energies in the associates Cu(acac)_2_···1,4‐DITFB (‐31 kJ/mol) and VO(acac)_2_···1,4‐DITFB (−28 kJ/mol, see Table 1),

**Table 1:** Bond energy (*E_b_*) in (M(acac)_2_(**L**) complexes, (I···N) or (I···O) halogen bonded molecular associates and lattice stabilization energies (*E*_latt_) of respective crystals and co‐crystals (see Tables S1, S4 for more data and details).

Compound	*E*_latt_, kJ/mol^[^ ^a^ ^]^	*E_b_* (M─N) in M(acac)_2_(L) and *E_b_* (I···N) or (I···O) in XB associates, kJ/mol	Contribution of XB to *E*_latt_ *_,_* (CE‐B3LYP / DGDZVP), %
Cu(acac)_2_(**dmap**)	188 ^HF^	84^[^ ^c^ ^]^	N/A
Cu(acac)_2_(**hmta**)	N/A*	81^[^ ^c^ ^]^	N/A
Cu(acac)_2_(**dabco**)	N/A**	81^[^ ^c^ ^]^	N/A
Cu(acac)_2_(**dpe**)	N/A**	68^[^ ^c^ ^]^	N/A
Cu(acac)_2_(4.4′‐**bipy**)	N/A*	71^[^ ^c^ ^]^	N/A
Cu(acac)_2_·1,2‐DITFB	N/A***	32^[^ ^b^ ^]^	N/A
Cu(acac)_2_·1,3‐DITFB	N/A***	30^[^ ^b^ ^]^	N/A
Cu(acac)_2_·1,4‐DITFB	242 ^HF^	31^[^ ^b^ ^]^	N/A
VO(acac)_2_·1,4‐DITFB	N/A	28^[^ ^b^ ^]^	N/A
VO(acac)_2_ **py**	179 ^HF^	69^[^ ^c^ ^]^	N/A
VO(acac)_2_ **dmap**	214 ^HF^	91^[^ ^c^ ^]^	N/A
**dmap**·1,4‐DITFB^[^ ^13^ ^]^	204	46^[^ ^b^ ^]^	22
**dpe**·1,2‐DITFB	210	25^[^ ^b^ ^]^	23
**dpe**·1,3‐DITFB	221	24^[^ ^b^ ^]^	21
**dpe**·1,4‐DITFB	247	27^[^ ^b^ ^]^	21
**dpe**·1,4‐Br_2_TFB	205	17^[^ ^b^ ^]^	17
**dpe**·1,4‐I_2_Ph	197	17^[^ ^b^ ^]^	15
**bipy** ·1,4‐DITFB	218	33^[^ ^b^ ^]^	21
**hmta**·1,4‐DITFB	204	31	32
1,4‐DITFB	102	N/A	N/A
**dabco**·1,4‐DITFB	208	37^[^ ^b^ ^]^	39
Py‐B(C_6_F_5_)_3_	186 (B‐N)	128^[^ ^b^ ^]^	N/A

*Note*: Note that for E_latt_ computations the experimental geometry from SC‐XRD is used, while for E_b_ – the optimized one. Additional and quite significant destabilization of XBs in real solutions arises from thermal and solvation effects.^[^ ^72^, ^86^ ^]^

N/A – not applicable in this case or *the lattice energy cannot be reliably calculated in Crystal Explorer because the structure is polymeric, **disordered or ***exist only in bimolecular model simulation.

^[a]^ Calculated in Crystal Explorer 21.5 using CE‐B3LYP DGDZVP, unless indicated otherwise (e.g., ^HF^ (HF 3–21G));

^[b]^ M062X def2TZVPD

^[c]^ MO6 DEF2SVP

Variation of XB‐donor co‐formers and stoichiometry, when 1:1, 1:2 and 5‐fold excessive amounts of C_6_F_5_I, 1,2‐ and 1,3‐isomers of DITFB were taken in place of 1,4‐DITFB, shows no signs of interaction with [M(acac)_2_(L)] (M = Cu, VO, L = amine) and only respective starting compounds were identified after slow evaporation of the reaction mixtures.

Reactions of [Cu(acac)_2_]_2_(**dpe**) (Figure S6) with the less electrophilic 1,4‐dihalo‐benzenes: 1,4‐dibromo‐tetrafluorobenzene (1,4‐Br_2_TFB), and 1,4‐diiodo‐benzene (1,4‐I₂Ph) show reasonable dependence on the polarization of halogen atom.^[^ ^66^ ^]^ The non‐fluorinated 1,4‐I_2_Ph, with iodides attached to a less electron‐withdrawing benzene ring, expectedly does not co‐crystallize with Cu(acac)_2_ and shows no reactivity toward Cu(acac)_2_(**dpe**). In contrast, 1,4‐Br_2_TFB, with two bromines attached to a more electron‐withdrawing tetrafluorobenzene ring, co‐crystallizes with [Cu(acac)_2_]_2_(**dpe**) to form known Cu(acac)₂·1,4‐Br_2_TFB (1:1),^[^ ^64^ ^]^ but require modified conditions, compared to the readily reactive, highly polarized 1,4‐DITFB.

It should be noted that in the absence of strong lattice stabilization, Jahn–Teller distortion, or trans‐influence, the XB‐donor co‐formers may compatibly coexist with axial N‐ and O‐donor ligands in transition metals β‐diketonate complexes. The influence of the steric factors of the β‐diketonate ligand on the structure of copper(II) β‐diketonate co‐crystals with iodofluorobenzenes was investigated by Bokach, Kukushkin, et al.^[^ ^67^ ^]^ They reported a co‐crystal of polymeric bis[1‐(tert‐butyl)butane‐1,3‐dionato]copper(II) (dioxane) with 1,3,5‐triiodotrifluorobenzene (1,3,5‐TITFB), which is stabilized by I···O_diketon_ XBs (Figure S8a). This structure, where Jahn–Teller distorted copper(II) ^t−^Bu‐β‐diketonate dioxane complex, coexists with the XB‐co‐former, highlights the role of loose packing—enhanced solubility factor. The bulky *^t−^* Bu groups in dioxane complexes of copper(II) ^t−^Bu‐diketonate provide a less close packing than in corresponding Cu(acac)_2_ complexes, therefore allowing the stabilization of co‐crystal lattices Cu(II) dioxane ^t−^Bu‐diketonate complexes with iodofluorobenzenes.

Another example of a Jahn–Teller‐affected Cu(II) β‐diketonate, where the axial pyridine ligand is not eliminated upon co‐crystallization with XB‐donor was recently reported by Frontera, Carlucci, et al^[^ ^68^ ^]^ The XB‐donor, in this case, is redirected to an alternative XB acceptor – the lateral pyridine substituent of the (4‐pyridyl)butane‐1,3‐dionate ligand — (Figure S8b). And finally, the studies by Stilinović, Cinčić et al^[^ ^64^ ^]^ show that co‐crystallization of 1,4‐DITFB with octahedral Ni(II) (*d⁸*), Co(II) (*d⁷*), and distorted tetragonal pyramidal Zn(II) (*d¹⁰*) acetylacetonates, which are unaffected by Jahn–Teller or trans effects, does not displace the H₂O ligands. Instead, these reactions yield hybrid organo‐metallic co‐crystals: Ni(acac)_2_(H_2_O)_2_·1,4‐DITFB (1:1), Co(acac)_2_(H₂O)_2_·1,4‐DITFB (1:1), and Zn(acac)_2_(H_2_O)·1,4‐DITFB (2:1)—stabilized by I···O halogen bonds and H_aq_···O_acac_ hydrogen bonds (Figure S8c,d).

**Figure 6:**
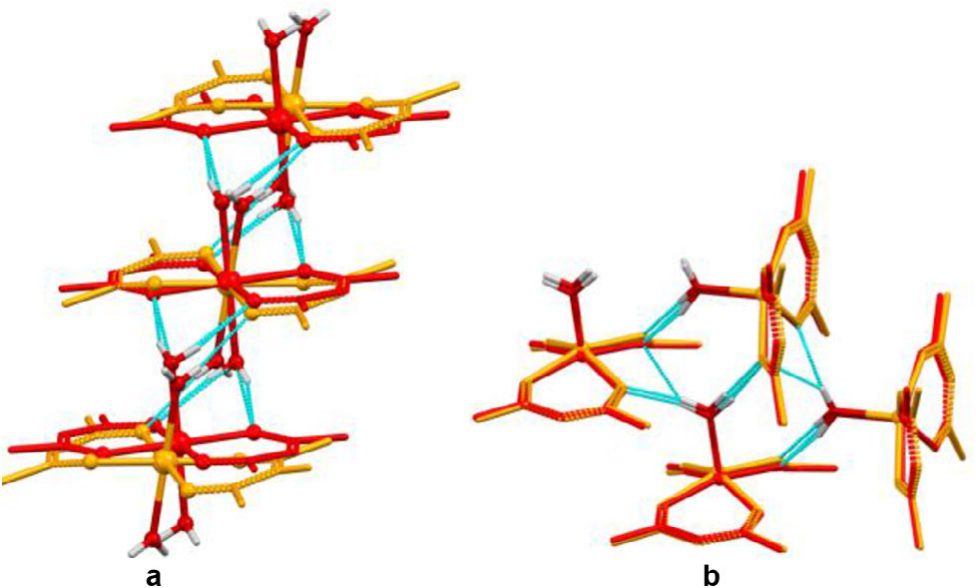
Overlay of the fragments of co‐crystal packings in native M(acac)_2_(H_2_O)_n_ (M = Co, Zn; *n* = 1, 2) aqua‐complex and their co‐crystal with DITFB, illustrating the conservation of hydrogen‐bonded supramolecular homosynthons: (a) Co(acac)_2_(H_2_O)_2_·(orange) and Co(acac)_2_(H_2_O)_2_·1,4‐DITFB (red); (b) Zn(acac)_2_(H_2_O) (orange) and Zn(acac)_₂_(H_₂_O)·1,4‐DITFB (red).^[^ ^64^ ^]^ For clarity, hydrogen atoms are omitted, except for those in the aqua‐ligands.

Apart from the electronic effects (preferable octahedral coordination geometry and absence of Jahn–Teller distortion for low‐spin *d^7^*, *d^8^, d¹⁰* metal ions), the other cause for the stability of M(acac)_2_(H_2_O) (M─Co, Ni, Zn) in the presence of 1,4‐DITFB is that the water ligands are integral parts of robust hydrogen‐bonded supramolecular homosynthon modules.^[^ ^69^ ^]^ These modules form the backbone of the native crystals Co(acac)_2_(H_2_O)_2_ (CODAAC05), Ni(acac)_2_(H_2_O)_2_ (ARUDUO), and Zn(acac)_2_(H_2_O)_2_ (ACAZM02), and in their corresponding co‐crystals with 1,4‐DITFB,^[^ ^64^ ^]^ this hydrogen‐bonded homosynthon structure is preserved^[^ ^70^ ^]^ (Figure 6, S9).

**Figure 7:**
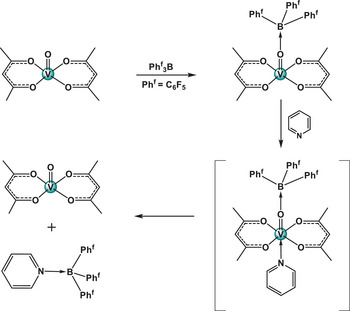
Showing the elimination of a pyridine ligand in the form of [Py‐B(C_6_F_5_)_3_] from [(py) (acac)_2_VO‐B(C_6_F_5_)_3_)].^[^ ^76^ ^]^

Taking into account that despite the destabilizing effects of solvation,^[^ ^71^, ^72^ ^]^ certain XBs are detectable in solutions^[^ ^27^, ^73^, ^74^, ^75^ ^]^ we can assume that the expected [M(acac)_2_(**L**)·1,4‐DITFB] (M = Cu, VO; L = **dmap**, **dpe**, **bipy**) structure, containing both the amine ligand and 1,4‐DITFB, may exist as an unstable intermediate, producing M(acac)_2_·1,4‐DITFB and L·1,4‐DITFB (Figures 4, 5). This tentative mechanism is somewhat similar to the well‐established elimination of a pyridine ligand in the form of a [py‐B(C₆F₅)₃] complex from [(py) (acac)₂VO‐B(C₆F₅)₃)]^[^ ^76^ ^]^ (Figure 7).

**Figure 8:**
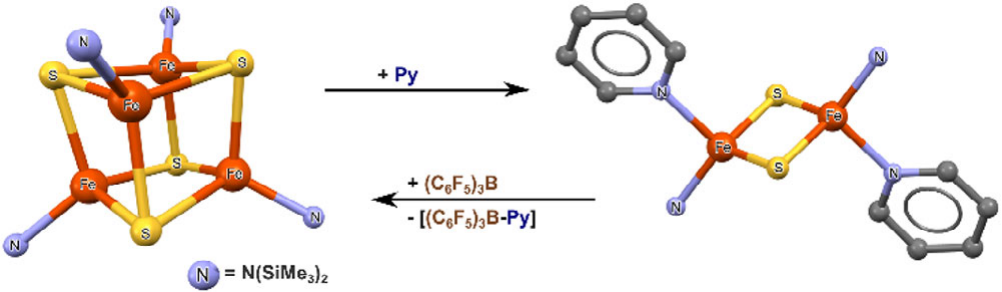
Showing the structural interconversion between [Fe_4_S_4_(N(SiMe_3_)_2_)_4_ cubane and dimeric Fe_2_S_2_(N(SiMe_3_)_2_) clusters.^[^ ^77^ ^]^ Only the cluster cores are shown for clarity, omitting hydrogens, counter‐ions, solvate molecules, and organic substituents in N(SiMe3)2 ligands.

A similar displacement of pyridine ligands from metal complexes by the strong Lewis acid B(C_6_F_5_)_3_, in the form of a [B(C_6_F_5_)_3_·py] complex, has been effectively used by Tatsumi et al.^[^ ^77^ ^]^ to demonstrate the structural interconversion between Fe_4_S_4_(N(SiMe_3_)_2_)_4_ cubane and dimeric Fe_2_S_2_(N(SiMe_3_)_2_) clusters (see Figure 8).

One definitely should keep in mind that the [B(C_6_F_5_)_3_·py] complex represents a stronger type of bonding (128 kJ/mol) than any XB associate in this work (see Table 1) or other exemplary strong I···N XBs.^[^ ^53^, ^78^, ^79^ ^]^ Unlike XB associates, the [B(C_6_F_5_)_3_·py] complex is thermally stable, reliably detectable in solutions by NMR and its molecular ions can be detected in mass spectra.^[^ ^80^ ^]^ Nevertheless, this prompts the potential preparative use of 1,4‐DITFB as a reagent for the elimination of N‐ligands in certain cases and proposes a detailed look at the energy of XBs and crystal lattices in the present case.

### 2.2 Computations of Supramolecular and Crystal Energetic

The calculated bond energies (the energy required to break the bond, *E*_b_, usually is a positive value) of the amine ligand (L) in its optimized complexes with M(acac)_2_(L) and L·1,4‐DITFB using DFT methods align well with the relative basicity of the respective amines (Table 1). In most cases, the energy of amine displacement in M(acac)_2_(L) is on average 30–40 kJ/mol higher than in L·1,4‐DITFB or M(acac)_2_·1,4‐DITFB. Even considering that the interaction of M(acac)_2_(L) with 1,4‐DITFB finally yields both L·1,4‐DITFB and M(acac)_2_·1,4‐DITFB (Figures 3, 4), the calculated enthalpy (Δ*H*) of the model reaction for Cu(acac)_2_(**dmap**) is + 6.2 kJ/mol, what is not quite in favor of **dmap** ligand displacement (Equation 1, Tables 1, S1): 

(1)

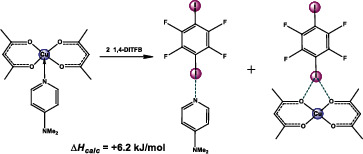




Since the XB alone cannot break this M─N coordination bond, additional factors such as molecular symmetry and lattice stabilization energy^[^ ^81^ ^]^ must be considered.

In general, the relative thermodynamic stability of the co‐crystal (Δ*E*_latt_) arises from the replacement of weaker intermolecular interactions with stronger bonds (in this case ─XBs), yielding a more stable co‐crystal lattice than the parent crystals. Quantitatively the Δ*E*_latt_ can be evaluated as the difference between the lattice energy (*E*_latt_) of the co‐crystal *A_n_B_m_* and parent crystals A and B^[^ ^82^ ^]^:

(2) $$\Delta E_{\mathrm{latt}}\left(A_{n}B_{m}\right)=E_{\mathrm{latt}}\left(A_{n}B_{m}\right)-\left[nE_{\mathrm{latt}}\left(A\right)+mE_{\mathrm{latt}}\left(B\right)\right]$$

Using this approach and periodic DFT, Taylor and Day evaluated the Δ*E*_latt_ for a set of 35 XB‐assisted organic co‐crystals as −12.6 kJ/mol on average and −26 kJ/mol at maximum.^[^ ^82^ ^]^ Such Δ*E*_latt_ value is sufficient to tip the scale in our Cu(acac)_2_ **dmap** / 1,4‐DITFB system (6.2 kJ/mol, Equation 1, S1) towards the formation of Cu(acac)_2_·1,4‐DITFB and **dmap**·1,4‐DITFB co‐crystals.

Our computations of *E*_latt_ based on Crystal Explorer HF/3–21 G energy model^[^ ^83^ ^]^ also highlight the higher thermodynamic stability of XB‐assisted co‐crystals of Cu(acac)_2_·1,4‐DITFB and **dmap**·1,4‐DITFB, compared to the lattices of their parent crystals (Table 1, Equation S1).

It should be mentioned that the total contribution of strong intermolecular interactions (like hydrogen bonds^[^ ^84^ ^]^) to *E*_latt_ typically does not exceed 50%. Respectively, the other influences, often broadly referred to as “packing factors”, contribute at least half to the stability of the resulting co‐crystals. Therefore, we evaluated the contribution of XB‐stabilized intermolecular interactions to *E*_latt_ of the resulting XB‐assisted co‐crystals of M(acac)_2_ and L with halobenzenes as the percentage of Σ*E*_total_ for the manually picked intermolecular interaction, which can be obviously considered as XB‐driven. On average, the energy of such intermolecular interactions provides a 21%–25% contribution to *E*_latt_ for the co‐crystals with fluorinated iodo‐XB donors and pyridyl XB‐acceptors. Weaker XB‐donors like 1,4‐DBrTFB and 1,4‐C_6_H_4_I_2_ provide an expectedly lower contribution to the *E*_latt_ of their co‐crystal with **dpe** (17% and 15% respectively) than 1,4‐DITFB (21%).

In the L·1,4‐DITFB (L = **dmap**, **dpe**, **bipy**) co‐crystals the energies of π···π interactions are comparable to those of I···N XBs, diminishing the relative impact of the latter. This effect is mostly pronounced in **dmap**·1,4‐DITFB, where the strongest **dmap**···1,4‐DITFB interaction^[^ ^50^ ^]^ contributes only a modest 22% to the *E*_latt_ (Table 1).

In the case of non‐aromatic amines, such as **dabco** and **hmta**, which cannot provide π–π stacking interactions, a markedly higher contribution of XB interactions is observed (39% and 32% respectively).

Another result which cannot be attributed to XB bonding energy is that calculated energies of the I···N**_dpe_** and I···O_M(acac)_ halogen bonds in DITFB···**dpe** and DITFB···Cu(acac)_2_ associates, fall within a narrow range around 25 ± 2 kJ/mol and 31 ± 1 kJ/mol for all three 1,2‐, 1,3‐, and 1,4‐DITFB isomers (see Table 1), yet only the 1,4‐DITFB is reactive towards Cu(acac)_2_(**dpe**).

**Figure 9:**
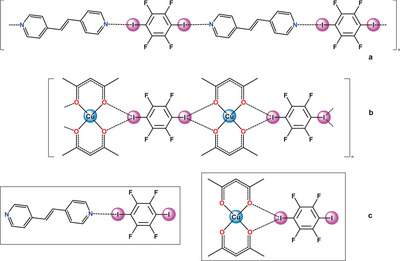
(a) Fragments of supramolecular polymeric chains (a) [M(acac)_2_·1,4‐DITFB]_n_ and (b) [**dpe**·1,4‐DITFB]_n_ stabilized by I···O and I···N XBs and (c) isolated bimolecular associate 1,4‐DITFB· ·(**dpe**) featuring only one I···N XB.

Therefore, we further analyzed the contribution of rather entropic factors, such as molecular and crystal symmetry to the lattice stabilization.^[^ ^85^ ^]^. In the present case, the supramolecular association is enhanced by the ability of ditopic molecules (M(acac)_2_, **dpe**,‐**bipy**, **dabco**, **hmta**, and 1,4‐DITFB) to chain up. Since the ditopic molecules of Cu(acac)_2_, **dpe**,‐**bipy**, **dabco**, **hmta** and 1,4‐DITFB can form supramolecular polymeric chains [Cu(acac)_2_⋅1,4‐DITFB]_n_ and [L⋅1,4‐DITFB]_n_ in the solid state (Figure 9a,b, S5, S10), the number of intermolecular XBs in a chain is doubled compared to simple bimolecular associates [Cu(acac)_2_⋅1,4‐DITFB] or [L⋅1,4‐DITFB] ((Figure 9c). Therefore, when forming a chain, even relatively weaker I···N XBs can outweigh the energetic benefit of the stronger ones. Considering the resulting chain symmetry, the entropic penalty is lower for supramolecular condensation of centrosymmetric molecules into the straight 1D‐chains (**dpe**,‐**bipy**, **dabco,** 1,4‐DITFB) rather than for non‐centrosymmetric molecules (**hmta**, 1,2‐DITFB, 1,3‐DITFB) into zig‐zags or more intricate architectures (Figure S10, Table 1).

We can briefly categorize the above tendencies into two major patterns:

(1) Higher stability of co‐crystal lattices formed by 1,4‐DITFB compared to those formed by 1,4‐DBrTFB and 1,4‐DIB. This aligns well with the dependence of XB strength on the relative polarizability of halogen atoms in an I > Br > Cl and the higher polarizing ability of perfluorinated benzenes compared to the non‐fluorinated.^[^ ^87^ ^]^ Experimental results agree with computational data, as we observe the precipitation of L·1,4‐dihalobenzene co‐crystals from M(acac)₂(**dpe**) solution only upon the addition of 1,4‐DITFB, but not with 1,4‐DBrTFB or 1,4‐DIB.

(2) Despite the similar energies of the respective bimolecular associates **dpe**·1,X‐DITFB (X = 2, 3, 4; *E*_b_ = 31 ± 1 kJ/mol, Table 1), the crystal lattices formed by centrosymmetric molecules of 1,4‐DITFB (**dpe**·1,4‐DITFB, *E*_latt_ 248 kJ/mol) are more stable compared to those formed by 1,2‐DITFB (210 kJ/mol) and 1,3‐DITFB (221 kJ/mol). This supports the role of molecular symmetry in crystal stabilization^[^ ^85^, ^88^, ^89^, ^90^ ^]^ and agrees with the experimental observation of only 1,4‐isomer of DITFB interacting with M(acac)₂(L).

**Figure 10:**
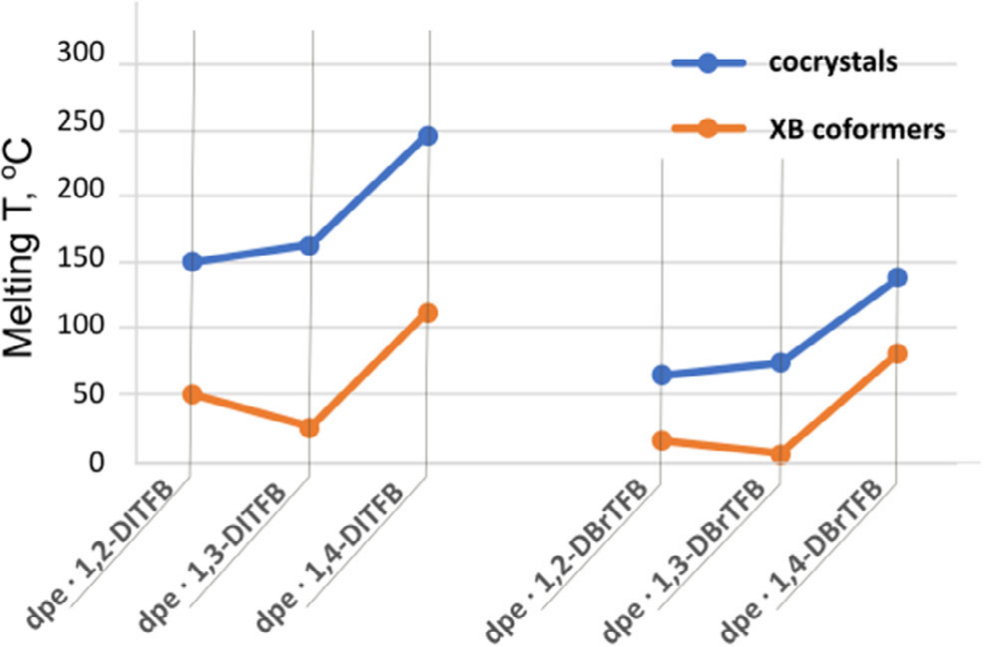
Melting points of **dpe**·dihaltetrafluorobenzene (1:1) co‐crystals (blue) and respective dihaltetrafluorobenzene co‐formers (orange).

Considering the melting point as a measure of lattice energy, we should note that the highly symmetric molecules of 1,4‐DITFB and 1,4‐DBrTFB result in higher‐melting native crystals and co‐crystals with amines, compared to their 1,2‐ and 1,3‐isomers. (Figure 10, Table S2).

Since the solubility has a reverse correlation with the melting point,^[^ ^91^, ^92^ ^]^ the higher melting point of 1,4‐DITFB and its co‐crystals aligns well with the lower solubility of its co‐crystals (Figure 10, Table S2), what makes them precipitate out from M(acac)_2_(**dpe**) / 1,4‐DITFB reaction mixture, in contrast to 1,2‐ and 1,3‐DITFB co‐crystals under the same conditions.

## 3 Conclusion

This study demonstrates the ability of XB‐donor co‐formers to induce ligand displacement in Jahn–Teller and trans‐influenced transition metal acetylacetonate complexes, leading to the formation of distinct co‐crystals. The driving force behind the unprecedented cleavage of the metal‐nitrogen coordination bond in M(acac)_2_(amine) (M = Cu, VO) upon the addition of an XB‐acceptor co‐former is the halogen bond stabilized lattice of the hybrid metal‐organic Cu(acac)_2_·1,4‐DITFB or the organic (amine)·1,4‐DITFB co‐crystals. The most efficient ligand displacement has been achieved with 1,4‐DITFB, which features the highly polarizable iodine functions attached to the C_6_F_4_ ring in the most symmetric manner, providing the strongest halogen bonding in combination with the highly efficient crystal packing. Computational analysis confirms that while the XB alone does not suffice to break metal–nitrogen coordination bonds, the formation of energetically favorable crystal lattices facilitates the observed reactivity. These results provide an insight into the new co‐crystallization‐assisted strategy (which may not be limited to XBs but applies to non‐covalent interactions in general), to control ligand displacement processes, and propose tracking the possible transient intermediates [M(acac)_2_(L)·1,4‐DITFB] as a promising direction for future investigation.

## Supporting Information

Supplementary file XB_versus_coordination_SI.pdf contains the experimental details of the co‐crystallization experiments, DFT computations, XRD and spectral, measurements, additional figures, tables and comments. The authors have cited additional references within the Supporting Information.^[^ ^66^, ^83^, ^93^, ^94^, ^95^, ^96^, ^97^, ^98^, ^99^, ^100^, ^101^, ^102^, ^103^, ^104^, ^105^, ^106^ ^]^

Deposition Number(s) 2400993 (for ([(Cu(acac)2)2(dpe)]dpe), 2400992 (for [(Cu(acac)2)3(hmta)2]n), 2411174 (for VO(acac)2(dmap)) contain(s) the supplementary crystallographic data for this paper. These data are provided free of charge by the joint Cambridge Crystallographic Data Centre and Fachinformationszentrum Karlsruhe Access Structures service.

## Conflict of Interests

The authors declare no conflicts of interest.

## Supporting information



Supporting Information
