# Supplementary material for: Halogen Bond‐Driven Ligand Displacement: Co‐Crystal Lattice Versus Coordination Bonds
Source: Chemistry. 2025 Apr 17;31(26):e202404784. doi: 10.1002/chem.202404784 (PMC12063052; doi:10.1002/chem.202404784)
Supplement: Supplementary file 1 — Supporting Information [file CHEM-31-e202404784-s001.pdf]

Halogen Bond-Driven Ligand Displacement: Co-crystal Lattice vs Coordination Bonds

Yury V. Torubaev*, Omer Shaashua, Savion Braunstein, Doron Pappo.

Department of Chemistry, Ben-Gurion University of the Negev

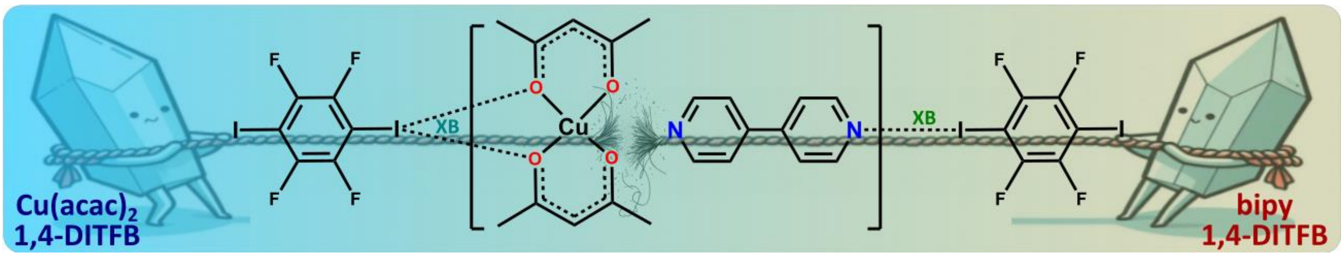

Table of contents.

	page
Experimental part	2
Interaction of M(acac)$_2$(L) with XB-donor halobenzenes	2
Figure S1. Solutions of (a) Cu(acac)$_2$(L)	2
Figure S2. Cu(acac)2(L) + 1,4-DITFB precipitation	3
Figure S3. Scheme for VO(acac)$_2$(L) + 1,4-DITFB	4
Computational details	5
UV-Vis Spectral Measurements	6
Figure S4. UV absorbance spectra.	6
Crystal Structure Analysis (XRD)	7
Figure S5. Crystal structure of [Cu(acac)$_2$)$_3$(μ$_3$-hmta)]$_n$	8
Figure S6. Crystal structure of [(Cu(acac)$_2$)$_2$(μ-**dpe**)]·**dpe**	9
Figure S7. Molecular structure of [VO(acac)$_2$(**dmap**)]	10
Figure S8. Examples of 1,4-DITFB β-diketonates cocrystals	11
Figure S9. Structural overlays	12
Figure S10. Crystal structures of L·1,X-DITFB (X=2,3,4) co-crystals	13
Figure S11. Experimental and simulated PXRD patterns	14
Figure S12. Experimental and simulated PXRD patterns	15
Table S1. Bond energies	16
Equitation S1.	18
Table S2. Melting points of L·halobenzene (1:1) co-crystals	19
Table S3. Crystal and structure refinement data	20
Figure S13. Structural overlay.	21
Figure S14. Structural overlay.	21
Figure S15. Structural overlay.	22
Figure S16. Structural overlay.	22
Table S4-S35. Atomic coordinates for the optimized structures	23-77
References	78

Experimental part

Materials and methods

Commercial solvents were dried and distilled prior to use. Commercial $Cu(acac)_2$, $VO(acac)_2$, **dpe**, **dmap**, **dabco**, **hmta**, 4,4'-**bipy**, 1,2-, 1,3, 1,4-DITFBs, 1,4-DBrTFB, C_6F_5I and 1,4-DIB were used without additional purification.

Interaction of $M(acac)_2(L)$ with XB-donor halobenzenes.

1.1. $Cu(acac)_2(L)$ (L = htma, dpe, bipy, dabco, dmap). General procedure

To a magnetically stirred 0.1 M ink-blue solution of $Cu(acac)_2$ in DCM (0.1 mL), 0.2 mL of a 0.1 M DCM solution of amine L was added. The color of the solution instantly turned to the lighter shades of blue (for L= htma, dpe, bipy, dabco) of blue-green (for L= dmap). The resulting clear solution was treated with 50 µL of a 0.1 M DCM solution of 1,4-DITFB. After 5 minutes, an additional 100 µL portion of the 0.1 M DCM solution of 1,4-DITFB was added.

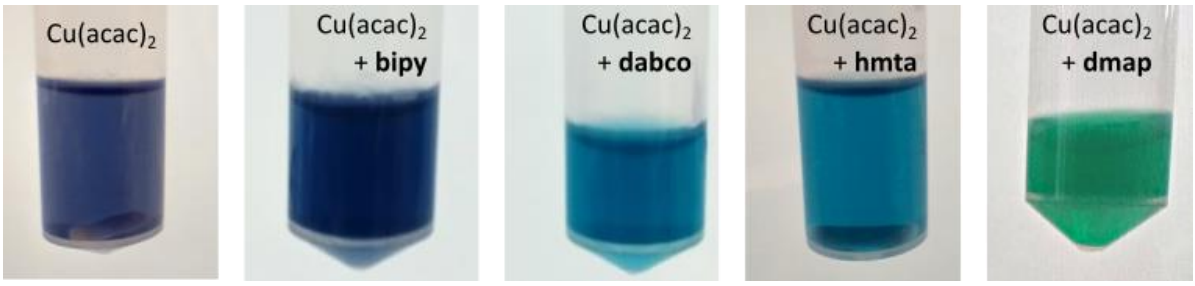

a b c d e

Figure S1. Solutions of (a) $Cu(acac)_2$ in DCM on addition of (b) **bipy**, (c) **dabco**, (d) **hmta** and (e) **dmap**.

1.1.1 Interaction of $Cu(acac)_2(L)$ with 1,4-DITFB. L = dmap, htma, dabco.

Following the general procedure, formation of characteristic blue micro-crystalline $Cu(acac)_2$·1,4-DITFB and respective color change to pale-blue was observed after the resulting reactions mixtures were kept undisturbed for 3 hours.

Addition of 0.5 ml of hexane (which is an antisolvent in this case) to the reaction mixtures allowed instant formation of characteristic blue micro-crystalline $Cu(acac)_2$·1,4-DITFB and respective color change to pale-blue.

Interaction of $Cu(acac)_2(L)$ with 1,4-DITFB. L = dpe, bipy.

Following the general procedure, an instant white precipitation (L·1,4-DITFB), and further formation of characteristic blue micro-crystals of $Cu(acac)_2$·1,4-DITFB and respective color change to light-blue was more pronounced for **dpe**, but also noticeable in case of **bipy**. Further formation of characteristic twinned blue $Cu(acac)_2$·1,4-DITFB co-crystals and decolorization of the solution to pale-blue was observed after the solutions were kept undisturbed for 3 hours.

Addition of 0.5 ml of hexane (which is an antisolvent in this case) to the reaction mixtures allowed instant formation of characteristic twinned blue micro-crystalline $Cu(acac)_2$·1,4-DITFB and respective color change to pale-blue.

PXRD analysis of the precipitates in $Cu(acac)_2$ - **dpe** - 1,4-DITFB system showed the mixtures of **dpe**·1,4-DITFB and $Cu(acac)_2$·1,4-DITFB phases, with the growing content of $Cu(acac)_2$·1,4-DITFB (from ~10% to ~50%) as the additional portion of 1,4-DITFB was added and the reaction mixture was allowed to stay undisturbed (Figure S11).

Interaction of $Cu(acac)_2$(L) with 1,4-DBrTFB. L = dpe, dabco

Following the general procedure for the preparation of **$Cu(acac)_2$(L)**, the resulting clear blue/green solution was treated with 50 µL of a 0.1 M DCM solution of 1,4-DBrTFB. After 5 minutes, an additional 100 µL portion of the 0.1 M DCM solution of 1,4-DBrTFB was added. Formation of characteristically twinned blue micro-crystals of $Cu(acac)_2$·1,4-DBrTFB (Fig. S2c) and respective color change to pale-blue was noticed in case of **dpe**.

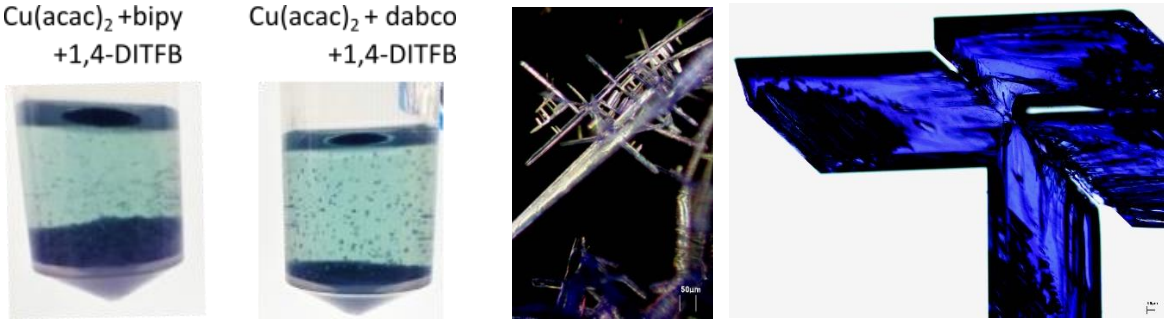

a b c d

Figure S2. $Cu(acac)_2$(L) + 1,4-DITFB in DCM. L = (a) dabco, (b)bipy; characteristic twinned co-crystals of (c) $Cu(acac)_2$·1,4-DBrTFB and of (d) $Cu(acac)_2$·1,4-DITFB.

Interaction of $Cu(acac)_2$(L) with other halobenzenes (1,2-DITFB, 1,3-DITFB, C_6F_5I, 1,4-DIB) (L = dmap, dabco, dpe).

Following the general procedure for the preparation of **$Cu(acac)_2$(L)**, the resulting clear blue/green solution was treated with 50 µL of a 0.1 M DCM solution of the respective halobenzene (1,2-DITFB, 1,3-DITFB, C_6F_5I, 1,4-DIB). No color change or precipitation was observed instantly and 5 hours later. Additional x5 portion of pure halobenzene was added and produce no change. The visually unchanged reaction mixtures were allowed to evaporate slowly for 48 h, resulting white materials and uniform blue or green crystals of the respective $Cu(acac)_2$(L) complexes.

Interaction of $VO(acac)_2$(L) with 1,4-DITFB. L = dmap, dpe.

To a magnetically stirred 0.1 M turquoise-blue solution of $VO(acac)_2$ in DCM (0.1 mL), 0.2 mL of a 0.1 M DCM solution of amine L was added. The color of the solution instantly immediately turned light-green for **dmap** and brownish-green in case of **dpe**. The resulting clear solution were treated with 50 µL of a 0.1 M DCM solution of 1,4-DITFB. After 5 minutes, an additional 100 µL portion of the 0.1 M DCM solution of 1,4-DITFB was added. Instant white precipitation, and respective color change to light green was noticed in case of **dpe**. In case of **dmap** no color change or precipitation was observed neither instantly nor 5 hours later.

Addition of the excessive amount of solid 1,4-DITFB (10 mg 0.025 mmol) to the $VO(acac)_2$(L) 1,4-DITFB reaction mixtures prepared as per general procedure results the white precipitate and significant lightening of the brownish green to green in case of **dpe**, and no evident changes in case of **dmap**.

The filtered reaction mixtures were allowed to evaporate slowly for 48h to produce characteristic yellow-green needles of $VO(acac)_2$·1,4-DITFB (1:1) [1].

PXRD analysis of the precipitates in $VO(acac)_2$ - **dpe** - 1,4-DITFB system showed predominantly the **dpe**·1,4-DITFB phase with a little (~5%) admixture of $VO(acac)_2$·1,4-DITFB phase (most likely resulting from the adsorbed solution residue).

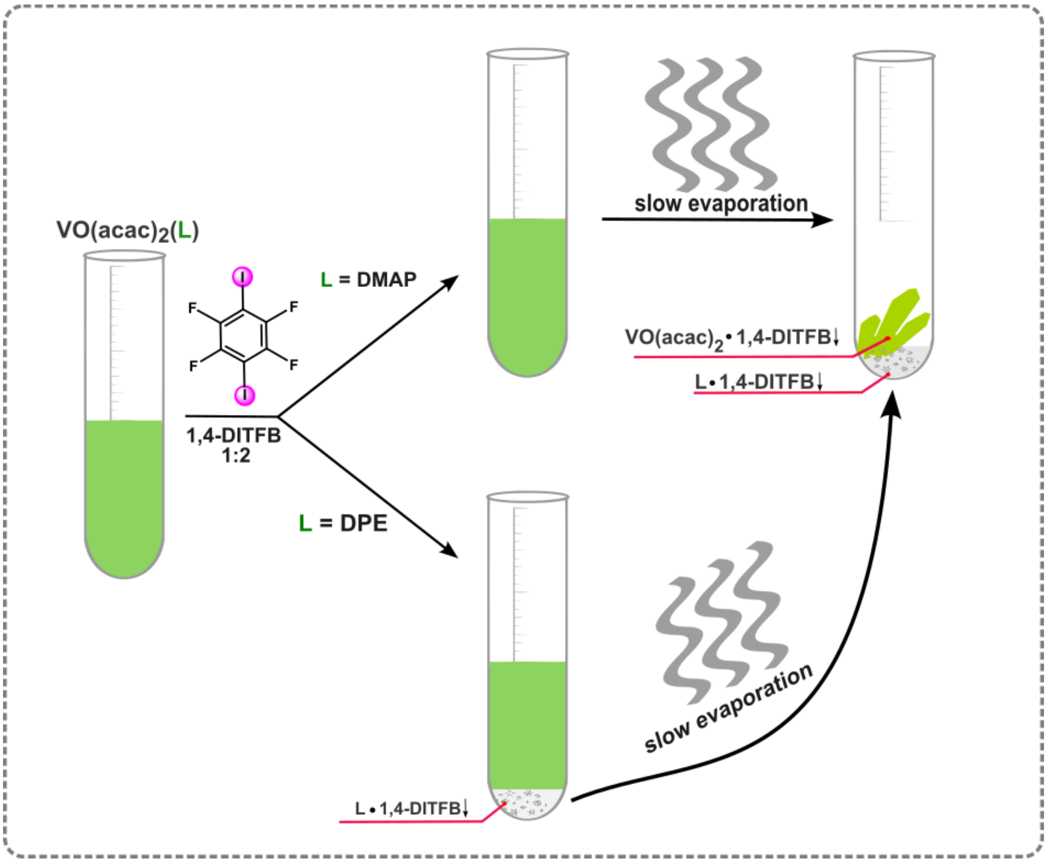

Figure S3. Interaction scheme for $VO(acac)_2(L)$ and 1,4-DITFB.

Computational details

Intermolecular interaction energy calculation and subsequent energy frameworks generation for crystals were performed using Crystal Explorer 21.5 (TONTO, HF 3-21G B3LYP DGDZVP) [2] for all unique molecular pairs in the first coordination sphere of a molecule (3.8Å), using experimental crystal geometries. HF 3-21G was the only energy model that allowed stable computation for triplet Cu(II) and V(IV) complexes in CE-TONTO. To validate this energy model, we calculated the lattice energies for singlet Pd(II) in Pd(acac)$_2$ and Pd(acac)$_2$ 1,4-DITFB and Al (III) in Al(acac)$_3$ both with HF 3-21G and B3LYP DGDZVP. The computation results (Table S1) show acceptable difference and the same trend of increasing the 1,4-DITFB co-crystal lattice stabilization, compared to the parent complex.

The enthalpies (ΔH) of I···N, I···O, metal-N and B-N bonds formation in amine-halobenzene, M(acac)$_2$-DITFB molecular associates, M(acac)$_2$(amine) and Py-B(C_6F_5)$_3$ complexes were calculated in Gaussian 16 [3] for optimized structures using MO6 and MO6-2X [4] functionals with Def2-SVP and Def2-TZVPD [5] basis sets respectively (Table S4).

Both MO6 and MO6-2X are functionals within the MO6 family, developed to suit different types of molecular systems[6]. MO6 is a general-purpose hybrid functional, designed for a broad range of systems, including main-group thermochemistry and transition metals, but may not be as optimized for highly noncovalent or dispersion-dominated interactions compared to MO6-2X. In turn, the MO6-2X functional which is a high-HF exchange (54%) version of MO6, emphasizing noncovalent interactions, especially van der Waals and halogen bonding. For the calculation of the ΔH of L ligand elimination from M(acac)$_2$(L), the MO6 functional was selected due to its better performance for a wide range of systems, especially those involving main-group elements and transition metals. For the halogen-bonded associates of halobenzene with M(acac)$_2$ and L, we applied the MO6-2X functional, which is known for its enhanced accuracy in non-covalent interactions like halogen bonding, π-π stacking etc.

Validation of this differential approach, through the calculation of the of bond energy (E_b) of L in M(acac)$_2$(L) and L·DITFB, using both M06 and M06-2X functionals, demonstrated a significant disparity in energy values computed for XB systems between these two functionals. Results from the M06 functional were systematically higher than those from M06-2X. For certain systems the relative M06 results were inconsistent with previous calculations for similar systems[7] and with general chemical reasoning. For example, the E_b of dissociation for [1,4-$I_2C_6H_4$·bipy] (30 kJ/mol) appeared higher than for the obviously stronger [1,4-$Br_2C_6F_4$·bipy] complex (26 kJ/mol). In contrast, for coordination-bonded systems, M06-2X and M06 provided similar or close results in most cases (see Table S1). However, for some M(acac)$_2$(L) complexes, the M06-2X ΔH of L ligand elimination yielded unrealistic values (>200 kJ/mol). Therefore, selecting the functionals from the same MO6 family, but using their specific versions (MO6 for coordination bonds in M(acac)$_2$(L); MO6-2X for the XB associates) provided a more reliable model for the analysis of the stability of these two systems.

Following the validation scheme[8], computations for non-optimized (SC-XRD experimental geometry) structures using different levels of theory (CE-HF 3-21G and MO6-2X/def2TZVPD) were compared with those for the optimized structures. We observed that both low- and high-level computations with only normalized hydrogen positions yield reasonable values comparable to those obtained from high-level optimized structures. These computations follow the same trend and suggest the same qualitative conclusions. This supports the the optimized model systems as valid representations of the real solid-state structures.

UV-Vis Spectral Measurements

The UV-Vis absorption spectra were recorded using the Varioskan LUX multimode microplate reader of ThermoFisher Scientific. The measurements were performed in the wavelength range of 200-1000 nm at room temperature. The instrument was equipped with a xenon flash lamp for the entire range. The samples were prepared by dissolving the compounds in methanol. The concentrations of the solutions were adjusted to $5{\cdot}10^{-3}$M. Liquid samples in standard 96-well polystyrene plates. Baseline correction was performed using the pure solvent (methanol) as a reference. All spectra were recorded at room temperature. The spectral data were processed using Spectragryph [9] software.

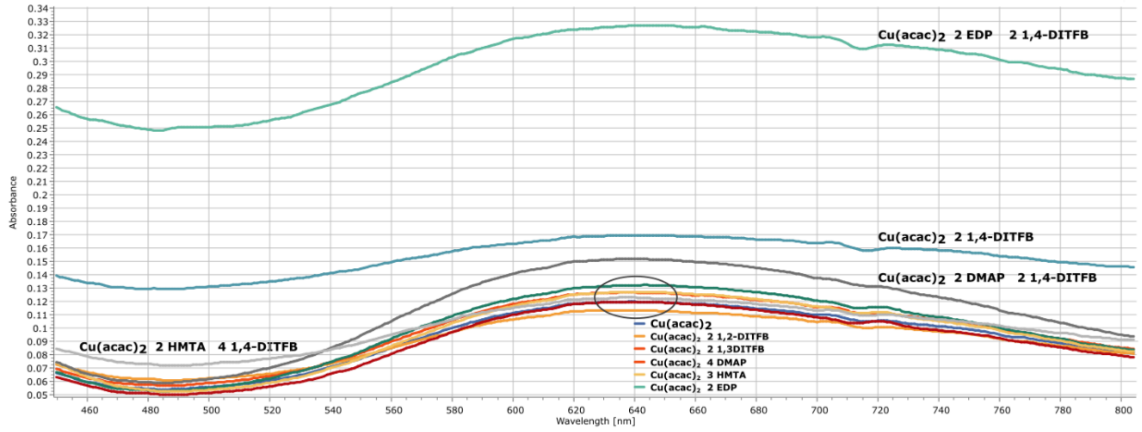

a

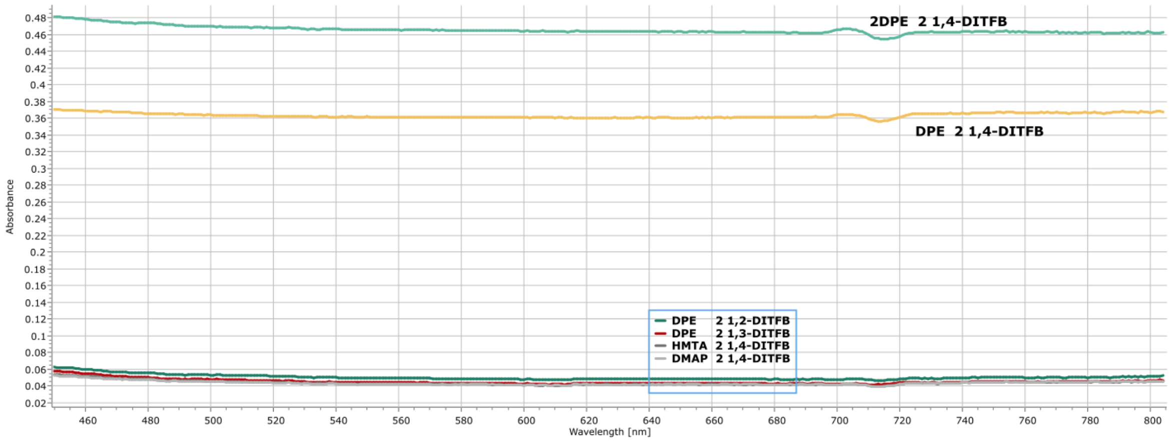

b

Figure S4. UV absorbance spectra in $5{\cdot}10^{-3}$ M methanolic solutions of (a) $Cu(acac)_2$ and its $Cu(acac)_2$(L) complexes mixe d with DITFBs in *d-d* transition range; (b) L and 1,X-DITFBs (X = 2, 3, 4). Notice the pronounced upshift of the baseline (indicating the turbidity of the liquid sample) for the systems containing **dpe** + 1,4-DITFB and $Cu(acac)_2$ + 1,4-DITFB.

Comments to Figure S4: Since the *d-d* transition peaks are low intensity (being formally forbidden in centrosymmetric coordination environment), broadened (due to ligand field distortions and variations in bond lengths owing to Jahn-Teller effect), the absorption bands are broad and less well-defined.) and their λ_{max} appear close to one another in a relatively narrow range of 630 – 640 nm, we are not attempting to make conclusive statements on the nature of respective interaction in the solution. However, one may notice that in case of 14-DITFB containing samples (namely: **dpe** 14-DITFB), $Cu(acac)_2$ 14-DITFB and $Cu(acac)_2$ **dpe** 14-DITFB)) the baseline appears significantly shifted upwards and additionally broadened. This is usually a result the turbidity caused by formation of some solid material. The turbidity leads to scattering of light, which is detected as additional "absorbance" in spectrum, even though it is not due to molecular absorption. In the present case the microcrystals are most likely

dpe·1,4-DITFB and $Cu(acac)_2$ 14-DITFB, formed according to the scheme:

$Cu(acac)_2$(**dpe**) + 2 1,4-DITFB = $Cu(acac)_2$·1,4-DITFB↓ + **dpe**·1,4-DITFB↓

Such a turbidity and resulting baseline upshift, is normally an undesirable effect of crystallization. However, in this case, it can serve as an indicator of co-crystallization—a process that cannot always be detected with the naked eye in diluted solution. With this in mind, we focused on the less pronounced baseline upshifts, which are clearly present in the $Cu(acac)_2$ **dmap** 14-DITFB system and less noticeable, but still evident, in the 460–560 nm range for the $Cu(acac)_2$ **hmta** 14-DITFB.

$Cu(acac)_2$(**dmap**) + 1,4-DITFB = $Cu(acac)_2$·1,4-DITFB↓ + **dmap**

$Cu(acac)_2$(**hmta**) + 1,4-DITFB = $Cu(acac)_2$·1,4-DITFB↓ + **hmta**

The absence of **dmap**·1,4-DITFB↓ and **hmta**·1,4-DITFB↓ precipitation here can be also assumed based by the absence of respective baseline upshifts in the UV spectra for L 1.4-DITFB (Figure S4b)

This is in good agreement with the observations for more concentrated solutions in DCM and formation of characteristic blue microcrystalline $Cu(acac)_2$ 1,4-DITFB precipitate upon the evaporation of MeOH from UV 96-well plate as well from the respective vials where the samples where stored.

Crystal Structure Analysis (XRD).

PXRD analysis was carried out on the fresh precipitates, separated by decantation using Rigaku XtaLAB Synergy (Cu Kα (1.5406 Å) and PANalytical Empyrean (Cu Kα (1.5406 Å; 2θ ranges of 5°−55°) X-ray diffractometers. Powder samples were mounted on an amorphous silicon holder (PANalytical Empyrean) or dispersed in paraffin oil and mounted on a loop (Rigaku XtaLAB Synergy) at room temperature. Diffraction data was analyzed using the GSAS software package [10] (ver. 5793, released 2024]. CIF files for the known structures were retrieved from CCDC CSD (version 2024).

SCXRD. Relevant crystallographic data and the details of measurements for single crystals of [$(Cu(acac)_2)_2$(**dpe**)]·**dpe**, [$(Cu(acac)_2)_3$(**hmta**)$_2$]$_n$ and $VO(acac)_2$(**dmap**) are given in Table S3 and the online supplementary (M_acac2_all.cif file).

Rigaku XtaLAB Synergy diffractometer equipped with graphite-monochromated Mo Kα radiation (0.71070 Å) and Cu Kα (1.5406 Å) were used for the cell determination and the intensity data collection. Data collection, reduction and analysis were performed with the CrysAlisPro software package (version 1.171.39.22a, Rigaku OD, 2018). was used for the cell determination and intensity data collection. Structures were solved by intrinsic phasing method and refined using least squares method for F^2 in anisotropic approximation in SHELXTL and Olex2 software [11],[12]. Atomic coordinates and other structural parameters**,** have been deposited with the Cambridge Crystallographic Data Centre (CCDC 2400993 ([$(Cu(acac)_2)_2$(**dpe**)]·**dpe**), 2400992 [$(Cu(acac)_2)_3$(**hmta**)$_2$]$_n$ and 2411174 ($VO(acac)_2$(**dmap**)).

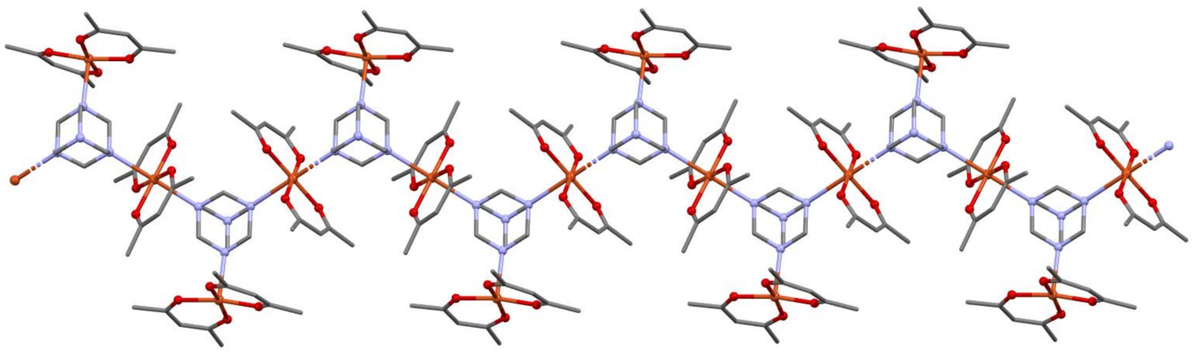

a

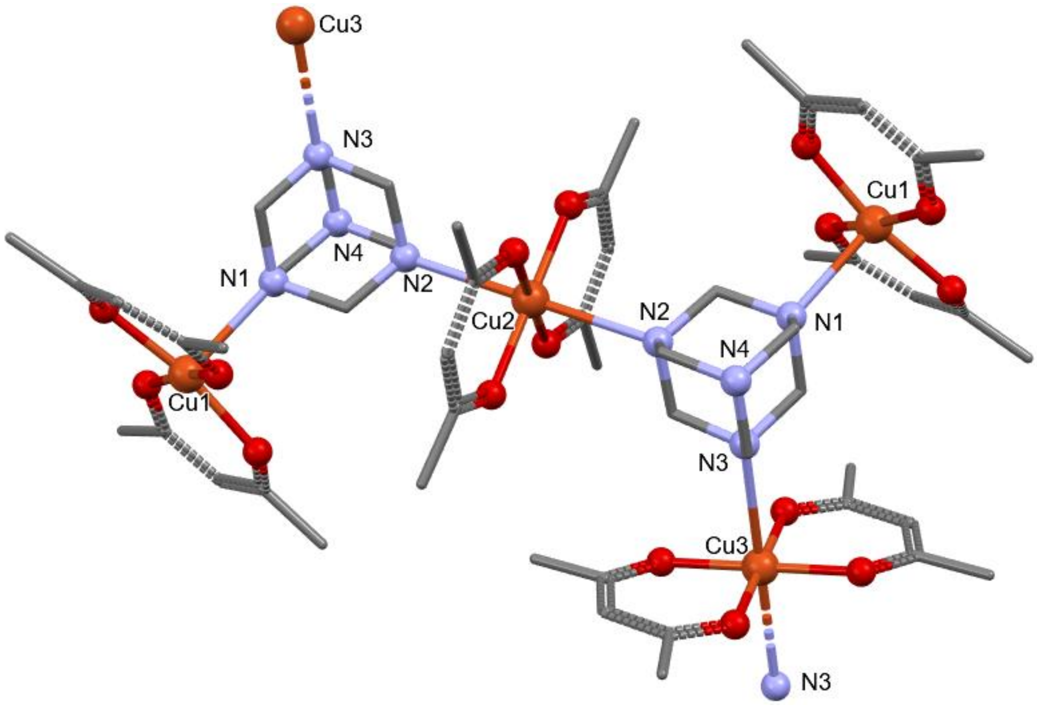

b

Figure S5. (a) Fragments of polymeric chains in the crystal packing [Cu(acac)$_2$)$_3$(μ^3-**hmta**)]$_n$ and (b) its structural unit. Hydrogen atoms are omitted for clarity. Selected distances, Å: N1 Cu1 2.360(2), N2 Cu2 2.520, Cu3 N3 2.632.

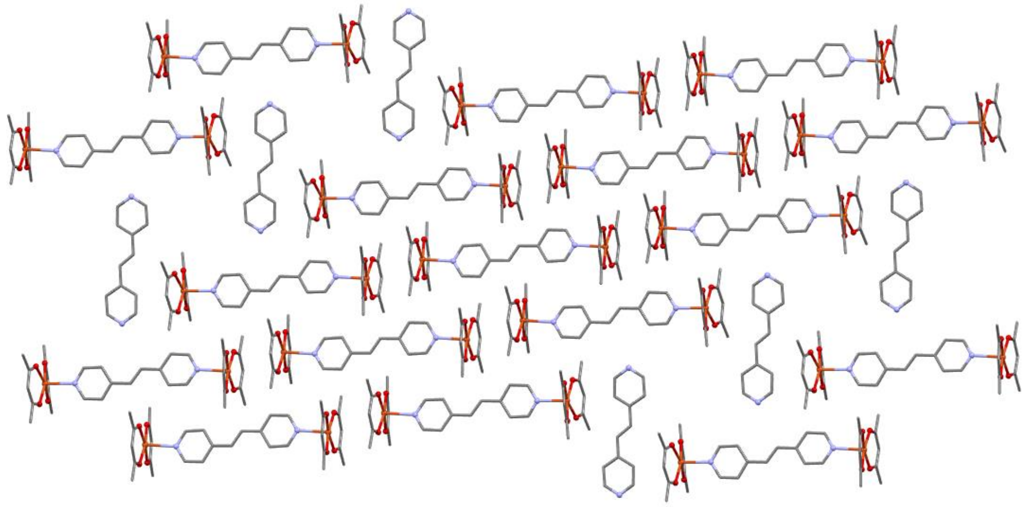

a

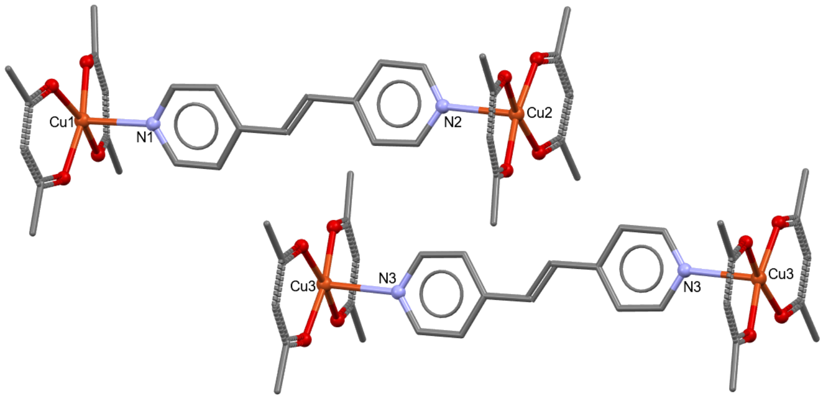

b

Figure S6. (a) Fragments of the crystal packing of [(Cu(acac)$_2$)$_2$(μ-**dpe**)]·**dpe** and (b) structure of two independent [Cu(acac)$_2$(**bipy**)]·molecules. Hydrogen atoms are omitted for clarity. Selected distances, Å: Cu1 N1 2.271(2), N2 Cu2 2.323(2), Cu3 N3 2.338(2), N3 Cu3 2.338(2).

Comments for Figures S5 and S6.

The trans influence is another significant factor that can further destabilize Cu-N bonding. For exam[le In polymeric Cu(acac)$_2$(**hmta**) (**hmta** = hexamethyltetraamine, Fig. 2. S3), which contains both 5-coordinate (CuO_4N) and 6-coordinate (CuO_4N_2) Cu centers, the elongation / weakening of the Cu-N distance from 2.362 Å (81 kJ/mol) in the tetragonal pyramidal CuO_4N fragment to 2.522 Å (66 kJ/mol, Table S1) for the axial Cu-N distances in the octahedral CuO_4N_2 fragment is illustrative. Another illustrative example is the formation of co-crystal of 5-coordinated Cu (II) complex in a co-crystal with **dpe** [(Cu(acac)$_2$)$_2$(μ-**dpe**)]·**dpe** (2:1) instead of coordination polymer [(Cu(acac)$_2$)(μ-**dpe**)]$_n$ (like in [(Cu(acac)$_2$)(μ-**bipy**)]$_n$ where Cu(II) is octahedral [13]). The **dpe** molecule here prefer to enter the crystal structure as an uncoordinated quest. Given the comparable energies of the Cu-N bonds in the optimized fragments of [(Cu(acac)$_2$)(**bipy**)$_2$] (71 kJ/mol) and [(Cu(acac)$_2$)(**dpe**)$_2$] (68 kJ/mol) and ~20 kJ/mol difference between Cu-N bonds energy in tetragonal CuO_4N pyramid [(Cu(acac)$_2$)(**dpe**)] (71 kJ/mol) and octahedral CuO_4N_2 in [(Cu(acac)$_2$)(**dpe**)$_2$] (47 kJ/mol. Table S1), this may serve as another example of packing factors dominating over Cu-N bond formation in a trans- and Jahn-Teller affected complex.

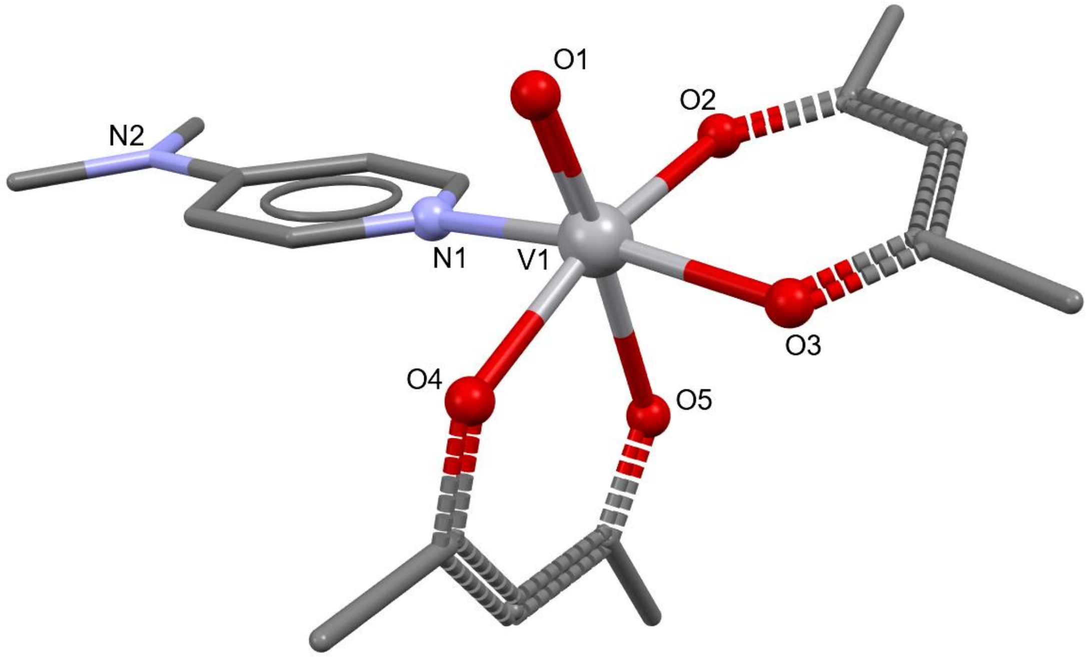

Figure S7. Molecular structure of [VO(acac)$_2$(**dmap**)].
Selected distances, Å: N1 V1 2.145(3), V1 O2 1.980(3), V1 O3 2.017(4), V1 O5 2.157(4), V1 O4 2.000(3).

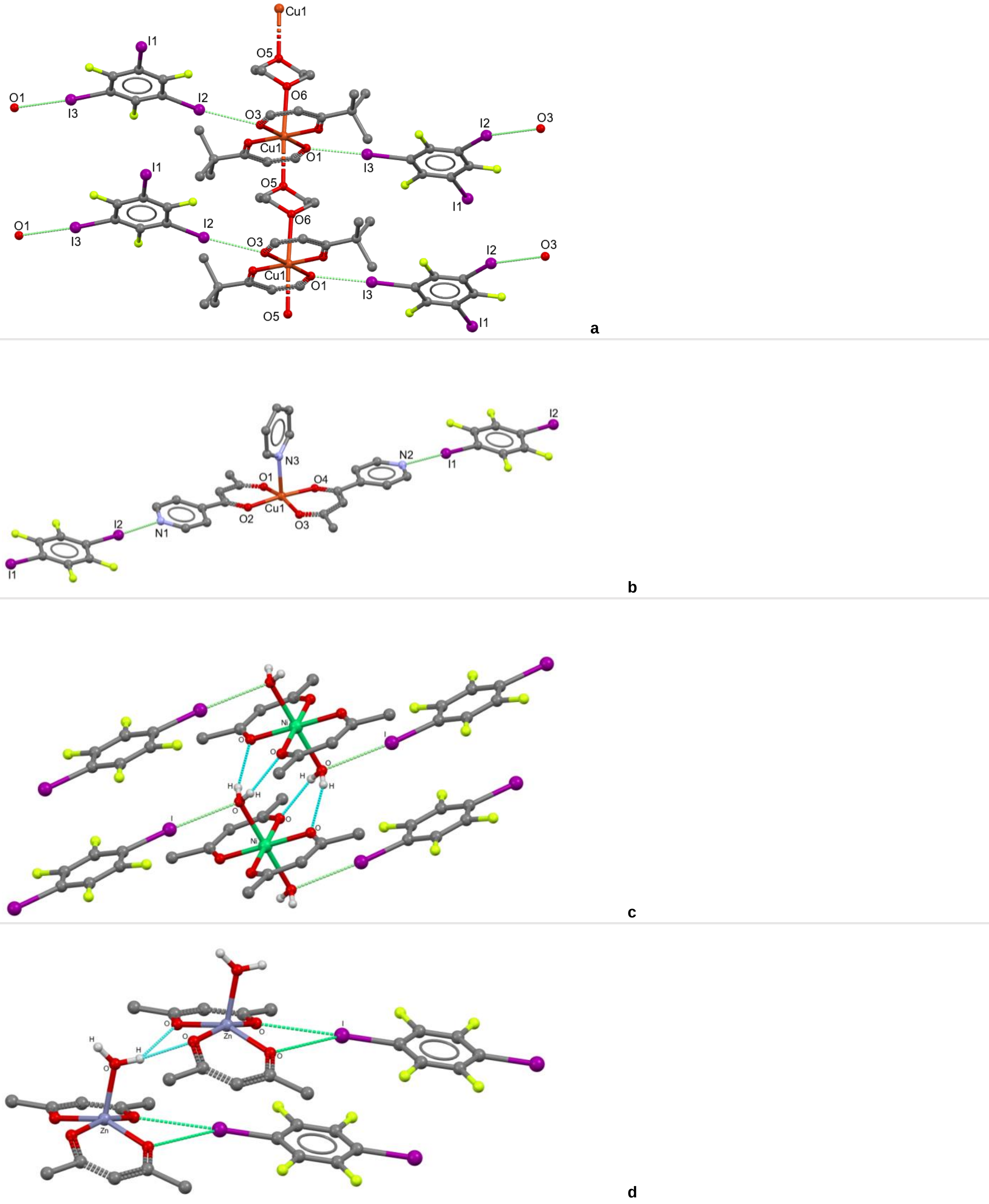

Figure S8. Known examples of co-crystals containing 1,4-DITFB and bis-β-diketonates complexes with O- and N-ligands. Fragments of co-crystal packings of (a) bis[1-(tert-butyl)butane-1,3-dionato]copper(II)(dioxane)·1,3,5-TITFB [14]; (b) (bis[1-(4-pyridyl)butane-1,3-dionato]copper(II)) [15], (c) Ni(acac)$_2$(H$_2$O)$_2$·1,4-DITFB and (d) Zn(acac)$_2$(H$_2$O)·1,4-DITFB[1] illustrating intermolecular I···O halogen bonds (light-green) and H···O hydrogen bonds (blue). Co(acac)$_2$(H$_2$O)$_2$ 1,4-DITFB (1:1) (SIXPUN) is isostructural with (a) Ni(acac)$_2$(H$_2$O)$_2$ 1,4-DITFB (1:1) (SIXPIB) and is not shown here. For clarity, hydrogen atoms are omitted, except for those in the aqueous ligands.

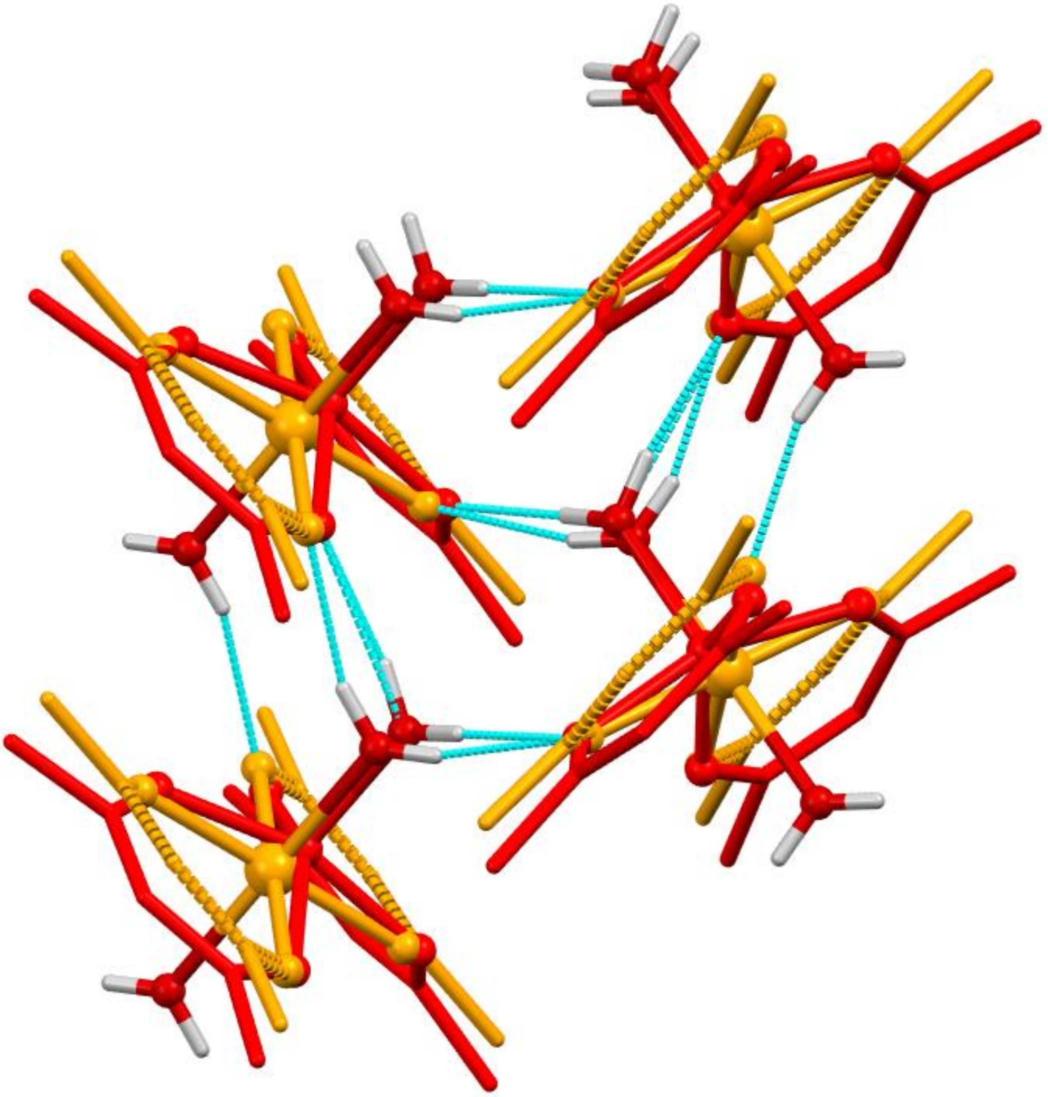

Figure S9. Overlay of the fragments of (a) $Co(acac)_2(H_2O)_2$·(CODAAC05) (orange) and $Zn(acac)_2(H_2O)$ (ACAZM02) (red) crystal packings, illustrating hydrogen bonded supramolecular synthons. For clarity, hydrogen atoms are omitted, except for those in the aqueous ligands.

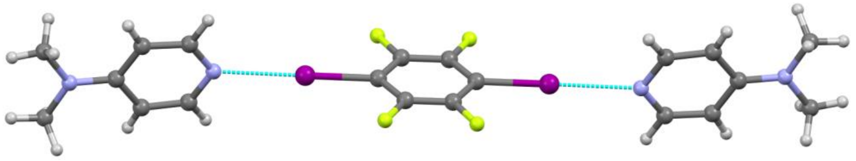

a

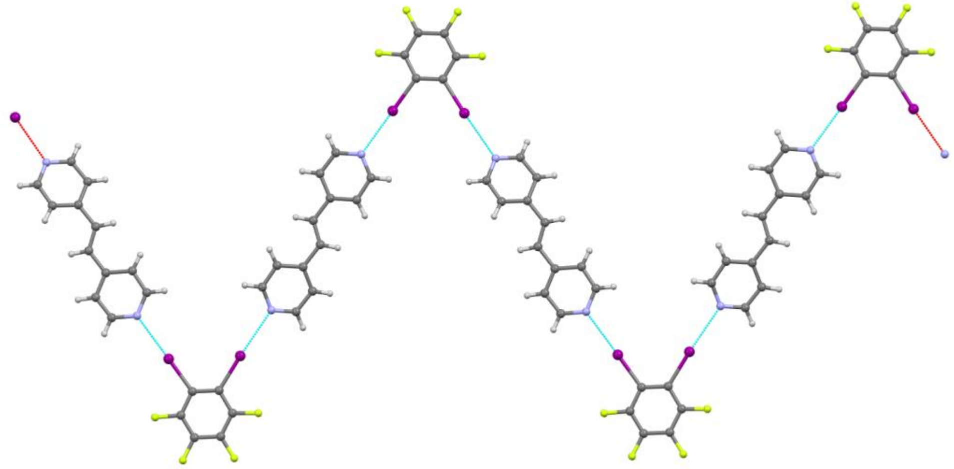

b

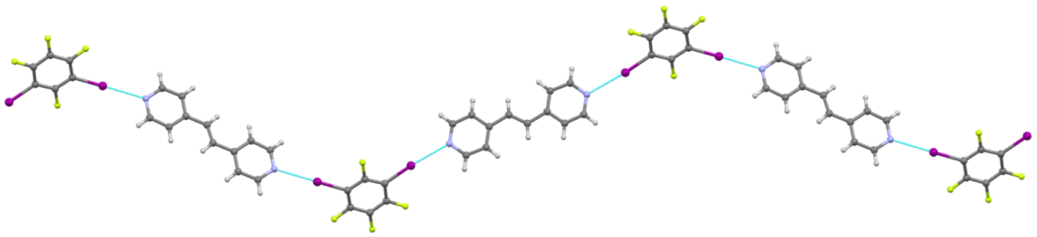

c

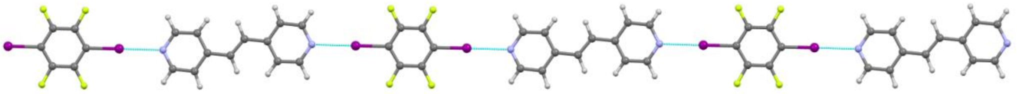

d

Figure S10. Fragments of crystal structures of **L**·DITFB co-crystals (with the respective CSD codes):
(a) **dmap**·1,4-DITFB (2:1) RUYHID ;
(b) **dpe**·1,2-DITFB (1:1) XEDYAK;
(c) **dpe**·1,3-DITFB (1:1) ZABWAE;
(d) **dpe**·1,4-DITFB (1:1) QIHCAL01.

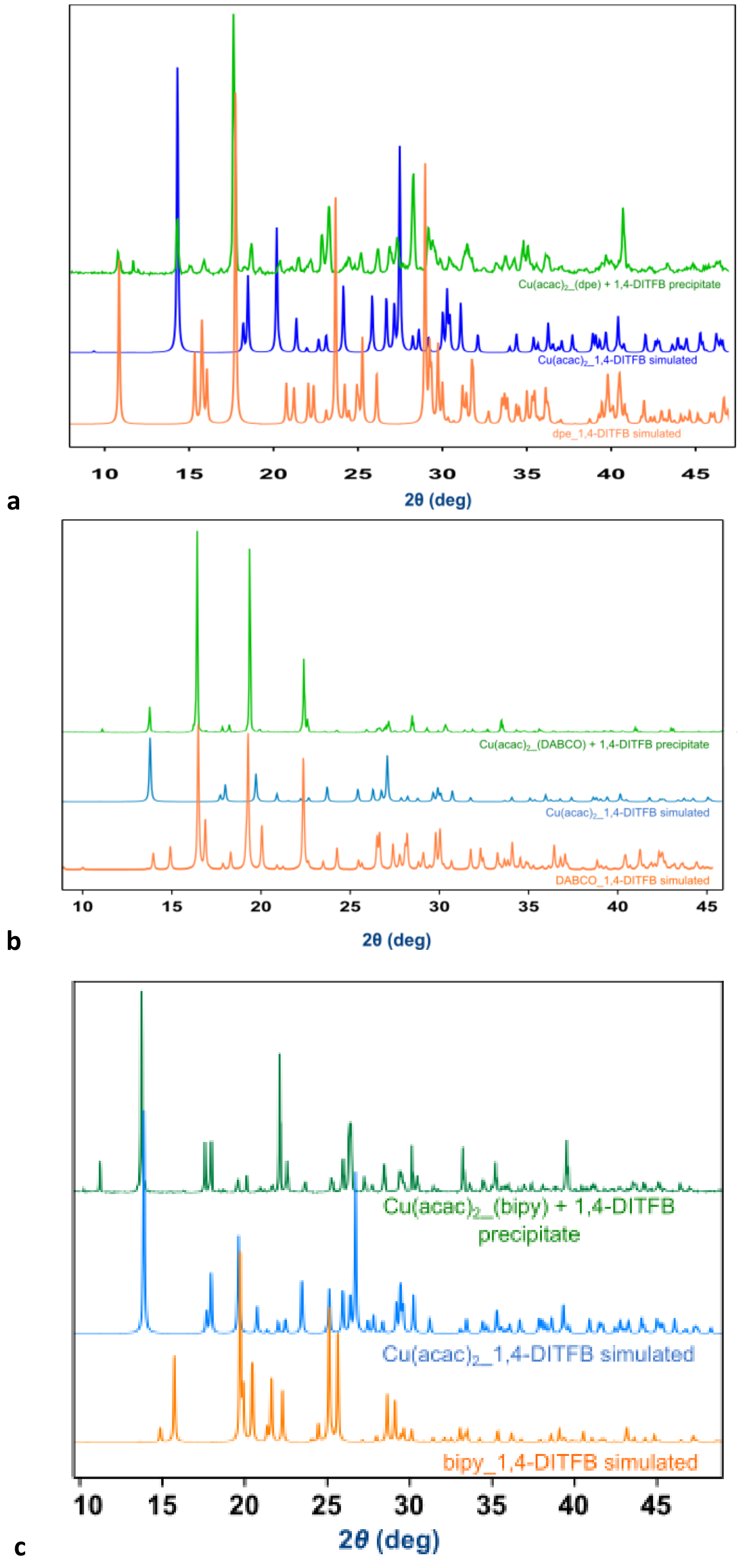

Figure S11. Experimental PXRD pattern of (a) Cu(acac)$_2$(**dpe**) + 1,4-DITFB precipitate compared to simulated Cu(acac)$_2$·1,4-DITFB and **dpe**·1,4-DITFB; (b) Cu(acac)$_2$(**dabco**) + 1,4-DITFB precipitate compared to simulated Cu(acac)$_2$·1,4-DITFB and **dabco**·1,4-DITFB; (c) Cu(acac)$_2$(**dipy**) + 1,4-DITFB precipitate compared to simulated Cu(acac)$_2$·1,4-DITFB and **bipy**·1,4-DITFB Depending on the relative solubility of Cu(acac)$_2$·1,4-DITFB and **L**·1,4-DITFB, the Cu(acac)$_2$(**L**) : 1,4-DITFB ratio and reaction time, different phase ratio may be observed.

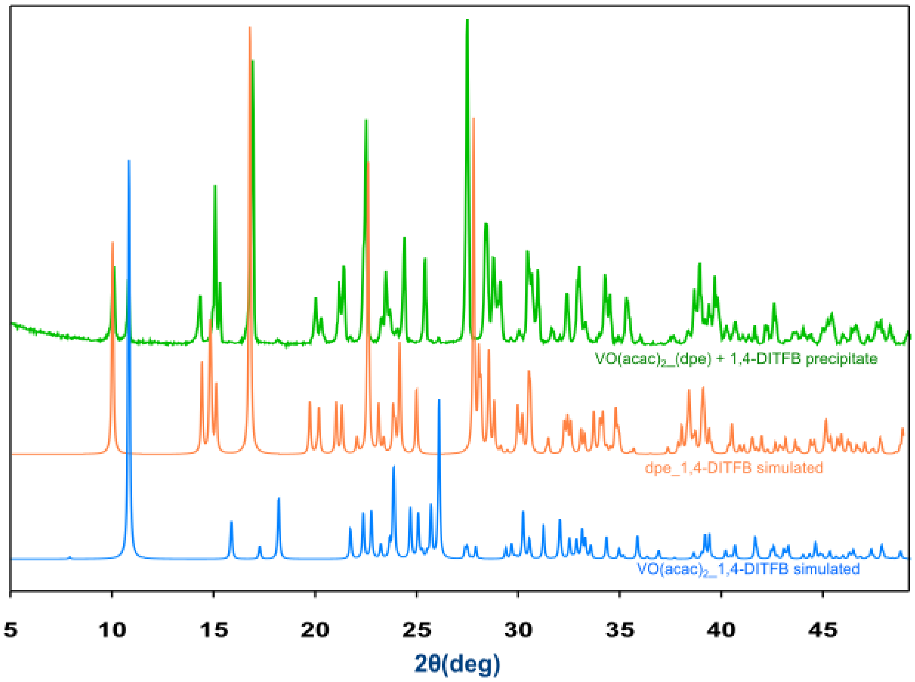

Figure S12. Experimental PXRD pattern of (a) $VO(acac)_2$(**dpe**) + 1,4-DITFB precipitate compared to simulated $VO(acac)_2$·1,4-DITFB and **dpe**·1,4-DITFB. Mostly the **dpe**·1,4-DITFB phase dominates at all conditions.

Table S1. Bond energies in M(acac)$_2$L complexes, halogen bonded molecular associates and lattice stabilization energies respective co-crystals. Binding energy (E_b) is calculated as ΔE of the dissociation reaction for the optimized structures, while interaction energy (E_i) is calculated for the experimental geometries [16].

	$E_{lattice}$ CE-HF / 3-21G, kJ/mol	$E_{lattice}$ CE-B3LYP/ DGDZVP kJ/mol	Interaction energy, E_i CE-HF / 3-21G, kJ/mol, and its contribution to E_{latt}, %	Interaction energy, E_i CE-B3LYP / DGDZVP kJ/mol, and its contribution to E_{latt}, %	Binding energy and ΔH M-N, E_b/ ΔH MO6 DEF2SVP kJ/mol	Binding energy M-N, E_b and ΔH M-N, E_b/ ΔH MO6X2 DEF2 TZVPD kJ/mol
			O---I	O---I	M-N<br>M= Cu, VO	M-N<br>M= Cu, VO
Pd(acac)$_2$ ACACPD01	167	-149	N/A	N/A	N/A	N/A
Pd(acac)$_2$ 1,4-DITFB (1:1) SESTIW02	259<br>128	-260<br>-130	30.7	30.4	39 / -33	25 / -26
Al(acac)$_3$ ALACAC28	143<br>Experimental 123 [17]	-140	N/A	N/A	N/A	N/A
Cu(acac)$_2$ ACACCU41	133<br>(experimental H_{subl} -127 [18])	*	N/A	N/A	N/A	N/A
Cu(acac)$_2$(**dmap**) ADAHIX	188	*	N/A	N/A	84 / -73	77 / -67
Cu(acac)$_2$(**hmta**)	polymeric	*	N/A	N/A	66 ** / -62<br>81 / --69	74 / -69
Cu(acac)$_2$(**dabco**)	disordered	*	N/A	N/A	81 / 76	76 / -70
Cu(acac)$_2$(**dpe**)		*	N/A	N/A	47 **<br>68 / -62	249
Cu(acac)$_2$(**bipy**) DEDJAX11	polymeric	*	N/A	N/A	71 / -64	65 / -58
Cu(acac)$_2$ 1,2-DITFB	N/A	N/A	N/A	N/A	45 / -39	32 / -27
Cu(acac)$_2$ 1,3-DITFB	N/A	N/A	N/A	N/A	43 / -38	30 / -25
Cu(acac)2 1,4-DITFB (1:1) (SESTAO02)	242	*	32<br>26%	*	43 / -41	31 / -28
VO(acac)$_2$	140	*	N/A	N/A	N/A	N/A
VO(acac)$_2$ 1,4-DITFB (1:1) SIXPOH	Disorder	*	N/A	*	41 / -36	28 / -28
VO(acac)$_2$**py** CUCWUS01	179	*	N/A	N/A	69 / -64	57 / -51

VO(acac)$_2$**dmap**	214 HF	*	N/A	N/A	91 / -80	76 / --63
			E_i N···I	E_i N···I	E_b/ ΔH N···I	E_b/ ΔH N···I
**dmap** 1,4-DITFB (2:1) RUYHID	177	-204	21.6<br>17%	33.2<br>22%	60 / -52	46 / --35
**dpe** 1,2-DITFB (1:1) XEDYAK	200	-210	19.6 / 21.3<br>21%	24.0 / 24.7<br>23%	40 / -34	25 / 20
**dpe** 1,3-DITFB (1:1) ZABWAE	218	-221	20.3 / 20.6<br>18%	23.2 / 24.0<br>21%	36 / -31	22
**dpe** 1,4-DITFB (1:1) QIHCAL01	236	-247	20.2<br>17%	26.0<br>21%	42 / -36	27 / -22
**dpe** 1,2-Br$_2$TFB (1:1)						
**dpe** 1,3-Br$_2$TFB (1:1) (IKUJED)						
**dpe** 1,4-Br$_2$TFB (1:1) IKUHUR01	207	-205	16.2<br>16%	17.4<br>17%	26 / -20	17 *** / --12
**dpe** 1,4-I2Ph (1:1) QIHBUE	192	-197	11.4<br>12%	15.3<br>15%	30 / -24	17 *** / -12
**bipy** 1,4-DITFB (1:1) QIHBEO02	-204	-218	18.3<br>18%	23.2<br>21%	44 / -38	33 / 28
**dabco**-1,4-DITFB (1:1) ISIHUN03	-184	-208	30.4<br>33%	40.2<br>39%	51 / -46	37 / --31
**hmta** 1,4-DITFB (1:1) QIHCOZ	-191	-204	23.6<br>25%	32.9<br>32%	45	31
1,2-DITFB		−96 [19]				
1,3-DITFB		−85 [19]				
1,4-DITFB (α form)	-82	−102 [19]				
1,4-DITFB (β form)		−90 [19]				
**py**-B(C_6F_5)$_3$ GIZYOE01		-186			128 / -121	133

N/A – not applicable or not available

*- unstable computation for CE-TONTO B3LYP / DGDZVP

** - E_b of one axial **L** molecule in octahedral [Cu(acac)$_2$(**L**)$_2$] complex and consequent E_b of the next axial **L** molecule in the resulting tetragonal prism [Cu(acac)$_2$(**L**)]. Notice that lower E_b(Cu-N) for the first **L** is in good agreement with its higher Jahn-Teller destabilization (see the experimental Cu-N distances in octahedral [Cu(acac)$_2$(**hmta**)$_2$] (2.632, 2.520 Å) and tetragonal prism [Cu(acac)$_2$(**hmta**)] (2.360Å). fragments of [(Cu(acac)$_2$)$_3$(μ-**hmta**)$_2$]$_n$ (Figure S5).

*** Comments to the Table S1.

1. Notice that all three 1,4-Br$_2$TFB, 1,4-I$_2$Ph, and 1,4-DITFB form XB-assisted polymeric chains with **dpe**. However, only the co-crystals of **dpe**·1,4-Br$_2$TFB and **dpe**·1,4-DITFB are isomorphic. In contrast, in the absence of π---π-hole and F---H interactions, the [1,4-I$_2$Ph·**dpe**]n chains adopt a typical fishbone packing pattern, indicative of simple close packing without significant intermolecular interactions [20, 21]. This comparison qualitatively highlights the strengthening of the lattice, complemented by the quantitative evaluation of E_{latt} (-197 kJ/mol, -205 kJ/mol, -248 kJ/mol) as the XB strength increases.

The ΔE_{latt} for the formation of **dmap**·1,4-DITFB (1:2) and Cu(acac)$_2$·1,4-DITFB (1:1) from 2 Cu(acac)$_2$**dmap** and 3 equivalents of ·1,4-DITFB (following the actual co-crystal stoichiometry) can be roughly assessed as:

2Cu(acac)$_2$**dmap** + 3DITFB = 2 Cu(acac)$_2$·DITFB + **dmap**·2DITFB

ΔE_{latt} = [2 E_{latt}(Cu(acac)$_2$·DITFB) + E_{latt}(**dmap**·2DITFB)] - [2 E_{latt}(Cu(acac)$_2$**dmap**) + 3E_{latt}(DITFB)] = [2x242 + 177] – [2x188 + 3x82] = 39 kJ/mol.

Equitation S1.

Table S2. Melting points of **L**·halobenzene (1:1) co-crystals and respective halobenzenes.

**L** -XB_{donor}	m.p. , °C	[Ref.]	respective XB_{donor}	m.p. , °C
**dmap** 1,4-DITFB (2:1)	158	[22]	1,4-DITFB	108-110
**dmap** 1,4-DIB (2:1)	122 – 124	[23]	1,4-DIB	130 – 132
**dpe** 1,2-DITFB (1:1)	147-148 (dec.)	[24]	1,2-DITFB	49-50
**dpe** 1,3-DITFB (1:1)	159 (dec.)	[25]	1,3-DITFB	25-26
**dpe** 1,4-DITFB (1:1)	236 - 240	[20]	1,4-DITFB	108-110
**dpe** 1,2-DBrTFB (1:1)	60 - 64	[20]	1,2-DBrTFB	14-16
**dpe** 1,3-DBrTFB (1:1)	70 - 73	[20]	1,3-DBrTFB	5-6
**dpe** 1,4-DBrTFB (1:1)	130 - 135	[20]	1,4-DBrTFB	78-80
**bipy** 1,2-DITFB (1:1)	138 - 140	[20]	1,2-DITFB	49-50
**bipy** 1,3-DITFB (1:1)	n/a		1,3-DITFB	25-26
**bipy** 1,4-DITFB (1:1)	180 - 182	[20]	1,4-DITFB	108-110
**bipy** 1,2-DBrTFB (1:1)	62 - 65	[20]	1,2-DBrTFB	14-16m
**bipy** 1,3-DBrTFB (1:1)	65 - 67	[20]	1,3-DBrTFB	5-6
**bipy** 1,4-DBrTFB (1:1)	110 - 115	[20]	1,4-DBrTFB	78-80

Table S3. Crystal and structure refinement data for [(Cu(acac)$_2$)$_2$(μ-**dpe**)]·**dpe**, [(Cu(acac)$_2$)$_3$(μ^3-**hmta**)$_2$]$_n$ and VO(acac)$_2$(**dmap**).

Identification code	[(Cu(acac)$_2$)$_2$(μ-**dpe**)]·**dpe**	[(Cu(acac)$_2$)$_3$(μ^3-**hmta**)$_2$]$_n$	VO(acac)$_2$(**dmap**)
CCDC #	2400993	2400992	2411174
Empirical formula	$C_{108}H_{128}Cu_6N_8O_{26}$	$C_{60}H_{60}N_{12}O_{12}Cu_3$	$C_{17}H_{24}N_2O_5V$
Formula weight	2335.42	1331.82	387.32
Temperature/K	293(2)	293(2)	293(2)
Crystal system	triclinic	triclinic	triclinic
Space group	P-1	P-1	P-1
a /Å	12.6127(4)	9.8619(6)	8.0592(6)
b/Å	14.3035(5)	11.0560(7)	11.5204(7)
c/Å	16.9514(7)	15.7424(9)	11.6647(8)
α/°	66.665(3)	83.626(5)	96.029(5)
β/°	89.366(3)	72.086(5)	109.863(6)
γ/°	89.679(2)	77.093(5)	108.851(6)
Volume/Å3	2807.82(19)	1590.31(18)	935.45(12)
Z	1	1	2
ρ_{calc} g/cm^3	1.381	1.391	1.375
μ/mm^{-1}	1.190	1.062	0.558
F(000)	1214.0	687.0	406.0
Crystal size / mm^3	0.76 × 0.24 × 0.11	0.29 × 0.23 × 0.21	0.548 × 0.194 × 0.137
Radiation	Mo Kα (λ = 0.71073)	MoKα (λ = 0.71073)	Mo Kα (λ = 0.71073)
2Θ range for data collection/°	6.568 to 57.998	6.48 to 61.05	3.826 to 51.992
Index ranges	-15 ≤ h ≤ 16, -18 ≤ k ≤ 16, -23 ≤ l ≤ 19	-12 ≤ h ≤ 12, -14 ≤ k ≤ 14, -18 ≤ l ≤ 19	-9 ≤ h ≤ 9, -14 ≤ k ≤ 14, -14 ≤ l ≤ 14
Reflections collected	26762	20329	10145
Independent reflections	12615 [Rint = 0.0383, Rsigma = 0.0594]	7679 [Rint = 0.0618, Rsigma = 0.0869]	3634 [Rint = 0.0558, Rsigma = 0.0484]
Data/restraints/parameters	12615/0/671	7679/0/377	3634/0/233
Goodness-of-fit on F^2	0.985	0.978	1.035
Final R indexes [I>=2σ (I)]	R1 = 0.0457, wR2 = 0.1169	R1 = 0.0490, wR2 = 0.1087	R1 = 0.0768, wR2 = 0.2165
Final R indexes [all data]	R1 = 0.0887, wR2 = 0.1378	R1 = 0.1008, wR2 = 0.1265	R1 = 0.0909, wR2 = 0.2303
Largest diff. peak/hole / e Å^{-3}	0.59/-0.23	0.32/-0.28	1.57/-0.41

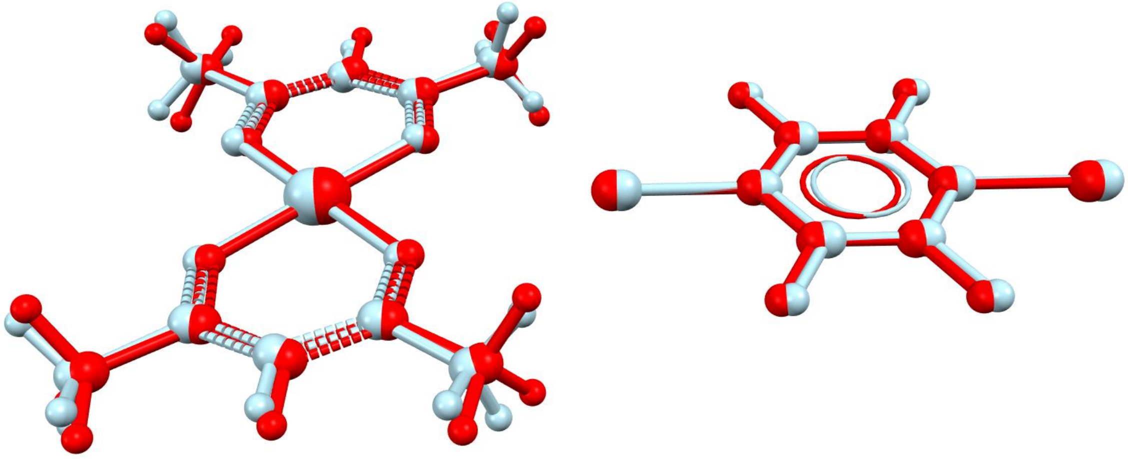

Figure S13. Structural overlay of optimized *in vacuo* (red) and experimental (SC-XRD, light-blue) geometry of the I···O$_{(acac)}$ XB associate of 1,4-DITFB and Cu(acac)$_2$.
Selected intermolecular distances (Å):
I···O 3.1652
I···O 3.1652
I1···O1 3.409
I1···O1A 3.409

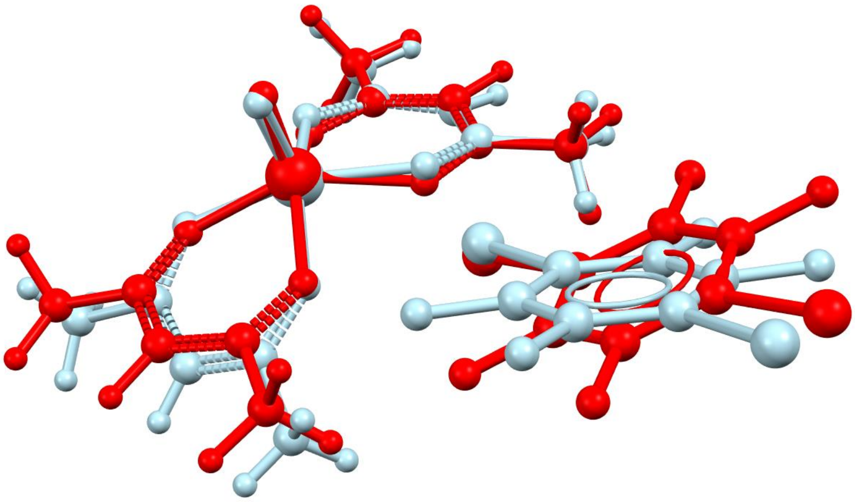

Figure S14. Structural overlay of optimized *in vacuo* (red) and experimental (SC-XRD, light-blue) geometry of the I···O$_{(acac)}$ XB associate of 1,4-DITFB and VO(acac)$_2$.
Selected intermolecular distances (Å):
I···O 3.1822
I···O 3.1776
I1···O1A 3.285(3)
I1···O3 3.352(3)

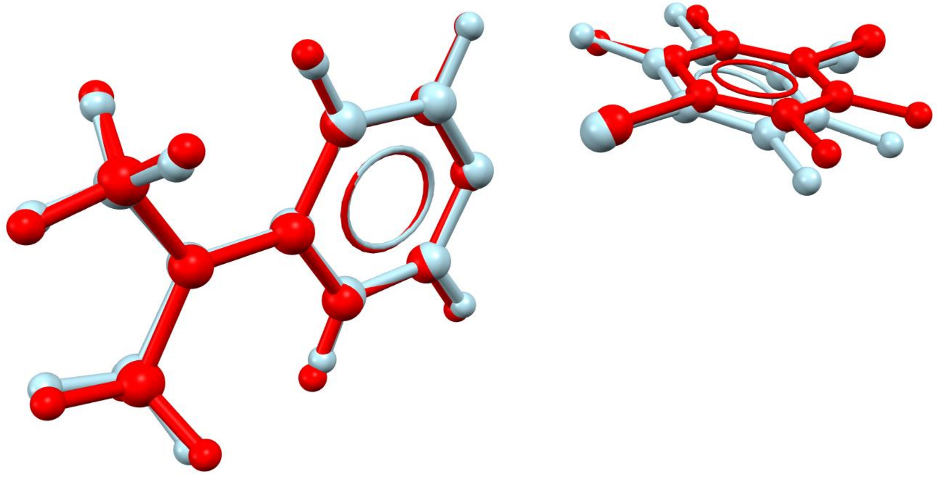

Figure S15. Structural overlay of optimized *in vacuo* (red) and experimental (SC-XRD, light-blue) geometry of the I···N XB associate of 1,4-DITFB and **dmap**. Selected intermolecular distances (Å):
I···N 2.8480
I1···N1 2.6630(6)

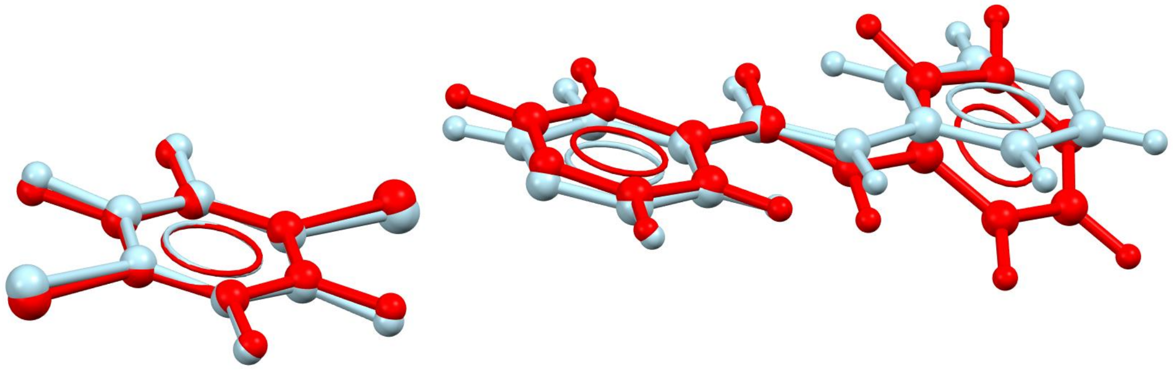

Figure S16. Structural overlay of optimized *in vacuo* (red) and experimental (SC-XRD, light-blue) geometry of the I···N XB associate of 1,4-DITFB and **dpe**. Selected intermolecular distances (Å):
I···N 2.9091
I1···N1 2.7804(9)

Table S4. Atomic coordinates for the optimized structure $Cu(acac)_2$

M062X def2TZVPD.

Cu	0.000000	0.000000	0.000000
O	1.645304	-1.026338	0.058404
O	-0.728737	-0.997257	1.496088
C	-0.171236	-1.935116	2.134959
C	1.936262	-1.961036	0.858562
C	1.106666	-2.442646	1.876843
H	1.471414	-3.247484	2.495306
C	-0.988523	-2.520446	3.254184
H	-1.239174	-1.725605	3.957549
H	-0.468614	-3.320690	3.774560
H	-1.926228	-2.896506	2.843548
C	3.296004	-2.573158	0.659392
H	3.360546	-2.959354	-0.358439
H	3.500921	-3.371058	1.368520
H	4.049797	-1.791086	0.756990
O	-1.645304	1.026338	-0.058404
O	0.728737	0.997257	-1.496088
C	0.171236	1.935116	-2.134959
C	-1.936262	1.961036	-0.858562
C	-1.106666	2.442646	-1.876843
H	-1.471414	3.247484	-2.495306
C	0.988523	2.520446	-3.254184
H	1.239174	1.725605	-3.957549
H	0.468614	3.320690	-3.774560
H	1.926228	2.896506	-2.843548
C	-3.296004	2.573158	-0.659392
H	-3.360546	2.959354	0.358439

H	-3.500921	3.371058	-1.368520
H	-4.049797	1.791086	-0.756990

MO6 DEF2SVP

Cu	0.000000	0.000000	0.000000
O	1.644094	-1.011002	0.046795
O	-0.738409	-0.982019	1.489236
C	-0.179663	-1.920361	2.127001
C	1.933633	-1.946045	0.847558
C	1.102525	-2.431747	1.869894
H	1.471731	-3.247252	2.496147
C	-0.994517	-2.504193	3.243102
H	-1.253789	-1.704755	3.955025
H	-0.479713	-3.315594	3.774629
H	-1.946878	-2.878068	2.835198
C	3.289647	-2.556237	0.649057
H	3.362076	-2.944119	-0.379280
H	3.508437	-3.362903	1.361322
H	4.054617	-1.768867	0.738811
O	-1.644094	1.011002	-0.046795
O	0.738409	0.982019	-1.489236
C	0.179663	1.920361	-2.127001
C	-1.933633	1.946045	-0.847558
C	-1.102525	2.431747	-1.869894
H	-1.471731	3.247252	-2.496147
C	0.994517	2.504193	-3.243102
H	1.253789	1.704755	-3.955025
H	0.479713	3.315594	-3.774629
H	1.946878	2.878068	-2.835198

C	-3.289647	2.556237	-0.649057
H	-3.362076	2.944119	0.379280
H	-3.508437	3.362903	-1.361322
H	-4.054617	1.768867	-0.738811

Table S5. Atomic coordinates for the optimized structure $VO(acac)_2$

M062X def2TZVPD

V	0.000000	0.000000	0.546154
O	-1.328458	-1.350070	-0.057091
O	0.000000	0.000000	2.099543
C	-3.244456	-0.000036	-0.325548
H	-4.307704	-0.000067	-0.505121
C	-3.327708	-2.507251	-0.443877
H	-2.882642	-3.056314	-1.274335
H	-4.384979	-2.343012	-0.634168
H	-3.204007	-3.117746	0.451434
C	-2.574184	-1.223098	-0.255647
C	-2.574249	1.223059	-0.255612
O	-1.328570	1.350092	-0.056811
C	-3.327790	2.507174	-0.444015
H	-3.204985	3.117275	0.451706
H	-4.384880	2.342865	-0.635247
H	-2.882054	3.056693	-1.273794
O	1.328458	1.350070	-0.057091
C	3.244456	0.000036	-0.325548
H	4.307704	0.000067	-0.505121
C	3.327708	2.507251	-0.443877
H	2.882642	3.056314	-1.274335
H	4.384979	2.343012	-0.634168
H	3.204007	3.117746	0.451434

C 2.574184 1.223098 -0.255647

C 2.574249 -1.223059 -0.255612

O 1.328570 -1.350092 -0.056811

C 3.327790 -2.507174 -0.444015

H 3.204985 -3.117275 0.451706

H 4.384880 -2.342865 -0.635247

H 2.882054 -3.056693 -1.273794

MO6 DEF2SVP

V 0.000000 0.000000 0.514299

O -1.328597 -1.350338 -0.057469

O 0.000000 0.000000 2.066224

C -3.252160 0.000205 -0.312644

H -4.330493 0.000312 -0.486110

C -3.328841 -2.507808 -0.426482

H -2.890411 -3.066369 -1.268490

H -4.402013 -2.357490 -0.602974

H -3.186298 -3.133678 0.468707

C -2.577619 -1.227182 -0.246742

C -2.577322 1.227423 -0.247150

O -1.328205 1.350318 -0.058155

C -3.328253 2.508187 -0.427103

H -3.185340 3.134275 0.467883

H -4.401510 2.358052 -0.603227

H -2.889946 3.066398 -1.269402

O 1.328597 1.350338 -0.057469

C 3.252160 -0.000205 -0.312644

H 4.330493 -0.000312 -0.486110

C 3.328841 2.507808 -0.426482

H	2.890411	3.066369	-1.268490
H	4.402013	2.357490	-0.602974
H	3.186298	3.133678	0.468707
C	2.577619	1.227182	-0.246742
C	2.577322	-1.227423	-0.247150
O	1.328205	-1.350318	-0.058155
C	3.328253	-2.508187	-0.427103
H	3.185340	-3.134275	0.467883
H	4.401510	-2.358052	-0.603227
H	2.889946	-3.066398	-1.269402

Table S6. Atomic coordinates for the optimized structure dmap

M062X def2TZVPD

N	-2.693043	0.000039	-0.000024
N	1.531939	-0.000031	0.000131
C	-1.978330	1.122003	0.000151
C	-0.597849	1.185812	0.000161
C	0.161213	-0.000070	0.000062
C	-0.597941	-1.185900	-0.000127
C	-1.978416	-1.121981	-0.000186
C	2.337468	1.198745	-0.000321
C	2.337685	-1.198669	0.000229
H	-2.543261	2.048737	0.000225
H	-0.142358	2.162544	0.000318
H	-0.142556	-2.162676	-0.000333
H	-2.543422	-2.048668	-0.000289
H	2.981454	1.221164	-0.885115
H	2.981269	1.221991	0.884581
H	1.735014	2.097450	-0.000824

H 2.982535 -1.220544 0.884397

H 2.980632 -1.222189 -0.885295

H 1.735440 -2.097501 0.001776

MO6 DEF2SVP

N -2.698277 0.000079 -0.000012

N 1.539038 -0.000063 0.000077

C -1.985990 1.123701 0.000094

C -0.599188 1.189505 0.000081

C 0.162581 -0.000142 0.000032

C -0.599377 -1.189690 -0.000062

C -1.986164 -1.123661 -0.000119

C 2.340080 1.195658 -0.000170

C 2.340538 -1.195498 0.000119

H -2.553036 2.065338 0.000105

H -0.131999 2.175446 0.000153

H -0.132392 -2.175713 -0.000150

H -2.553373 -2.065201 -0.000126

H 2.995619 1.230626 -0.890957

H 2.995476 1.231122 0.890700

H 1.733277 2.106365 -0.000485

H 2.996550 -1.229983 0.890570

H 2.995507 -1.230878 -0.891075

H 1.734174 -2.106471 0.000968

Table S7. Atomic coordinates for the optimized structure **hmta**.

M062X def2TZVPD

C	1.082385	-1.258896	-0.242664
H	1.658882	-1.435447	-1.153012
H	1.326013	-2.036536	0.484072
C	1.101056	1.075795	-0.667393
H	1.358756	2.055724	-0.260480
H	1.677525	0.911236	-1.579961
N	1.457358	0.045107	0.312502
N	-0.346527	-1.336700	-0.561532
C	-0.656253	-0.271424	-1.520070
C	-1.101003	-1.075698	0.668103
H	-1.721651	-0.306131	-1.756459
H	-0.867192	-1.852562	1.398866
H	-0.087170	-0.442795	-2.436091
H	-2.168801	-1.114289	0.443250
C	0.656811	0.272069	1.519516
C	-1.083232	1.258289	0.242487
H	0.912186	1.248734	1.935587
H	-0.837509	2.239718	0.653101
H	0.898785	-0.497666	2.255146
H	-2.150733	1.229422	0.014831
N	-0.327309	1.058254	-0.997527
N	-0.783190	0.233306	1.246740

MO6 DEF2SVP

C	-1.274740	-0.899797	0.604524
H	-1.206171	-1.911614	1.043110
H	-2.334204	-0.587615	0.635768

N	-0.828274	-0.947489	-0.785625
C	-0.918918	0.401337	-1.339439
H	-1.974337	0.728287	-1.329984
N	-0.112259	1.358295	-0.585808
C	-0.575518	1.352357	0.799719
H	-1.627118	1.689853	0.833016
H	0.028644	2.066198	1.388188
N	-0.476716	0.026193	1.404577
C	0.918727	-0.401362	1.339543
H	1.539902	0.292568	1.934105
H	1.012227	-1.407614	1.786333
C	0.575306	-1.352242	-0.799863
H	0.665111	-2.369171	-0.377134
H	0.933446	-1.386613	-1.844605
N	1.417698	-0.437184	-0.033143
C	1.274574	0.899920	-0.604461
H	-0.577811	0.386556	-2.390298
H	1.640824	0.890696	-1.646896
H	1.899757	1.608486	-0.031763

Table S8. Atomic coordinates for the optimized structure dabco.

M062X def2TZVPD

N	-1.278973	-0.004153	-0.002197
N	1.278988	0.004144	0.002106
C	-0.780060	1.183663	-0.697218
H	-1.146481	2.063853	-0.165364
H	-1.210092	1.200064	-1.700408
C	0.774854	1.157281	-0.745641
H	1.139510	1.079059	-1.771690
H	1.202896	2.062109	-0.310172
C	-0.772284	-1.198296	-0.680559
H	-1.133336	-1.179177	-1.710671
H	-1.201882	-2.076672	-0.195326
C	0.782439	-1.221727	-0.625282
H	1.146939	-2.069985	-0.042642
H	1.216058	-1.295484	-1.624336
C	-0.779779	0.007267	1.373934
H	-1.140598	-0.895603	1.870385
H	-1.214946	0.865304	1.889667
C	0.774813	0.071848	1.374848
H	1.134011	1.001612	1.820223
H	1.207919	-0.755230	1.940469

MO6 DEF2SVP

N	-1.264795	-0.000204	0.001013
N	1.264843	0.000117	-0.001014
C	-0.776247	1.365626	0.136750
H	-1.184391	1.792002	1.070337
H	-1.185763	1.967563	-0.693845

C	0.776050	1.365861	0.135342
H	1.184264	1.968116	-0.695765
H	1.185443	1.792218	1.068437
C	-0.777128	-0.565039	-1.250078
H	-1.186533	0.030104	-2.085655
H	-1.185960	-1.585650	-1.355894
C	0.775251	-0.565025	-1.251301
H	1.184191	-1.585580	-1.357691
H	1.183146	0.030158	-2.087616
C	-0.775173	-0.800884	1.115204
H	-1.183785	-1.822437	1.017943
H	-1.183271	-0.382698	2.052473
C	0.777189	-0.800465	1.114095
H	1.186549	-0.381721	2.050654
H	1.186112	-1.821903	1.016567

Table S9. Atomic coordinates for the optimized structure dpe

M062X def2TZVPD

N	4.701173	0.214056	-0.035214
C	1.921860	-0.198100	0.020367
C	3.853622	1.204314	0.239320
C	2.478323	1.054462	0.281901
C	4.173344	-0.981036	-0.274933
C	0.479758	-0.463328	0.026878
C	2.809818	-1.235252	-0.255747
N	-4.701168	-0.214109	-0.035249
C	-1.921858	0.198142	0.020353
C	-3.853572	-1.204355	0.239259
C	-2.478299	-1.054437	0.281921
C	-4.173377	0.981016	-0.274846
C	-0.479773	0.463359	0.026729
C	-2.809850	1.235267	-0.255707
H	4.299928	2.171757	0.441856
H	1.858287	1.904423	0.532415
H	4.873225	-1.780406	-0.492835
H	0.206574	-1.513284	-0.001439
H	2.441086	-2.232741	-0.459728
H	-4.299855	-2.171800	0.441839
H	-1.858229	-1.904336	0.532554
H	-4.873265	1.780362	-0.492821
H	-0.206577	1.513305	-0.001921
H	-2.441176	2.232782	-0.459662

MO6 DEF2SVP

N	4.716529	0.215275	0.000004

C	1.926213	-0.192511	0.000002
C	3.863514	1.238480	-0.000088
C	2.481080	1.096261	-0.000097
C	4.197774	-1.007947	0.000093
C	0.490395	-0.460072	0.000003
C	2.829053	-1.264180	0.000099
N	-4.716532	-0.215253	0.000017
C	-1.926211	0.192497	-0.000001
C	-3.863530	-1.238470	-0.000088
C	-2.481095	-1.096269	-0.000112
C	-4.197764	1.007960	0.000100
C	-0.490388	0.460049	-0.000018
C	-2.829038	1.264177	0.000093
H	4.303969	2.244464	-0.000167
H	1.846681	1.986996	-0.000184
H	4.908086	-1.845582	0.000173
H	0.222043	-1.524355	0.000038
H	2.459855	-2.294742	0.000179
H	-4.303999	-2.244448	-0.000188
H	-1.846709	-1.987013	-0.000228
H	-4.908064	1.845606	0.000187
H	-0.222033	1.524331	-0.000037
H	-2.459827	2.294735	0.000154

Table S10. Atomic coordinates for the optimized structure 4.4’-bipy

MO6 DEF2SVP

N	0.775133	-0.598223	3.432571
C	1.189351	0.498996	2.807049
H	1.780670	1.210666	3.398203
C	0.915074	0.780263	1.471737
H	1.296210	1.707919	1.038991
C	0.160977	-0.124373	0.713407
C	-0.271186	-1.277407	1.381019
H	-0.865048	-2.041229	0.874222
C	0.058749	-1.462264	2.720634
H	-0.281843	-2.367518	3.240379
C	-0.160977	0.124373	-0.713407
C	-0.915074	-0.780263	-1.471737
C	-1.189351	-0.498996	-2.807049
N	-0.775133	0.598223	-3.432571
C	-0.058749	1.462264	-2.720634
C	0.271186	1.277407	-1.381019
H	0.865048	2.041229	-0.874222
H	0.281843	2.367518	-3.240379
H	-1.780670	-1.210666	-3.398203
H	-1.296210	-1.707919	-1.038991

Table S11. Atomic coordinates for the optimized structure 1,2-DITFB

M062X def2TZVPD

F	-1.558099	2.698244	-0.000383
C	-1.507997	1.372383	-0.000225
C	-0.296548	0.698798	-0.000030
I	1.427529	1.856351	0.000191
I	1.431599	-1.853527	-0.000258
C	-0.294287	-0.698313	0.000406
F	-1.550644	-2.701084	0.000908
C	-1.504297	-1.374463	0.000299
C	-2.710363	-0.693632	0.000026
F	-3.853876	-1.358088	0.000078
F	-3.857426	1.349412	-0.000394
C	-2.712066	0.687559	-0.000198

Table S12. Atomic coordinates for the optimized structure 1,3-DITFB

M062X def2TZVPD

F	2.323279	2.267644	-0.000273
C	1.195328	1.574808	-0.000139
C	1.214006	0.189208	-0.000035
I	3.010389	-0.837750	-0.000045
F	0.001012	-1.809306	0.000817
C	0.000378	-0.482001	0.000154
I	-3.010520	-0.837737	-0.000194
C	-1.213676	0.188533	0.000488
C	-1.195106	1.574153	0.000143
F	-2.323453	2.266372	0.000582
F	-0.000607	3.597634	-0.000091
C	-0.000117	2.271915	-0.000054

Table S13. Atomic coordinates for the optimized structure 1,4-DITFB

M062X def2TZVPD

C	-1.402793	-0.000002	0.000201
C	-0.692387	1.189217	0.000068
C	-0.692382	-1.189218	0.000130
I	-3.470050	-0.000001	0.000518
F	-1.325548	2.355219	0.000130
F	-1.325541	-2.355220	0.000254
C	0.692480	1.189233	-0.000131
C	1.402835	0.000003	-0.000203
C	0.692485	-1.189231	-0.000069
F	1.325567	-2.355234	-0.000130
I	3.470017	0.000001	-0.000518
F	1.325559	2.355236	-0.000253

Table S14. Atomic coordinates for the optimized structure of pyridine

M062X def2TZVPD

N	-0.001290	-1.408876	-0.000071
C	1.135758	-0.720053	0.000374
C	1.192252	0.667627	-0.000191
C	0.001663	1.376263	-0.000038
C	-1.190923	0.669721	0.000064
C	-1.137661	-0.717289	-0.000290
H	2.049371	-1.304804	-0.000006
H	2.148263	1.173243	-0.000291
H	0.002329	2.458846	0.000298
H	-2.145508	1.178050	0.000550

H -2.051957 -1.300816 0.000433

MO6 DEF2SVP

N 0.000000 1.410501 0.000000

C -1.138296 0.723114 -0.000280

H -2.066878 1.309659 0.000595

C -1.196722 -0.670284 0.000075

H -2.161866 -1.184014 0.000067

C 0.000000 -1.380658 0.000000

H 0.000000 -2.474802 0.000000

C 1.138296 0.723114 0.000280

H 2.066878 1.309659 -0.000594

C 1.196722 -0.670284 -0.000075

H 2.161866 -1.184015 -0.000067

Table S15. Atomic coordinates for the optimized structure 1,4-Br2TFB

M062X def2TZVPD

C -1.398741 -0.000003 0.000209

C -0.692231 1.192454 0.000073

C -0.692231 -1.192455 0.000135

Br -3.266249 0.000000 0.000477

F -1.330615 2.352713 0.000139

F -1.330613 -2.352719 0.000262

C 0.692201 1.192402 -0.000135

C 1.398795 0.000003 -0.000208

C 0.692200 -1.192398 -0.000072

F 1.330665 -2.352719 -0.000139

Br 3.266225 -0.000001 -0.000477

F 1.330663 2.352726 -0.000262

Table S16. Atomic coordinates for the optimized structure 1,4-I2Ph

M062X def2TZVPD

C	1.380442	0.000002	0.000201
C	0.694220	-1.205146	0.000069
C	0.694217	1.205149	0.000132
I	3.469135	0.000000	0.000517
H	1.231549	-2.142982	0.000121
H	1.231536	2.142974	0.000233
C	-0.694212	-1.205189	-0.000132
C	-1.380381	-0.000002	-0.000202
C	-0.694214	1.205186	-0.000070
H	-1.231985	2.143730	-0.000122
I	-3.469126	0.000000	-0.000517
H	-1.231986	-2.143746	-0.000234

Table S17. Atomic coordinates for the optimized structure $Cu(acac)_2$dmap

MO6 DEF2SVP

C	-4.195881	-0.561684	1.553295
H	-4.375391	0.167849	0.747440
H	-4.472298	-0.059920	2.494259
H	-4.841605	-1.437833	1.404658
C	-2.731974	-0.901067	1.591260
C	-2.343389	-2.247359	1.469966
H	-3.126802	-2.998662	1.342055
C	-1.014354	-2.692070	1.590216
C	-0.736309	-4.168830	1.550828
H	-0.249229	-4.467382	2.492774

H	-0.012572	-4.377373	0.746753
H	-1.638247	-4.777215	1.398908
C	0.790189	-0.821854	-1.335491
H	1.427144	-1.484913	-0.734700
C	0.832306	-0.865234	-2.719226
H	1.508980	-1.568660	-3.207007
C	0.000000	0.000000	-3.465468
C	0.867686	-0.900818	-5.550651
H	0.650086	-1.960501	-5.322359
H	1.934880	-0.722031	-5.323565
H	0.730650	-0.759731	-6.630418
N	0.000000	0.000000	-0.644814
N	0.000000	0.000000	-4.832825
O	-1.956825	0.081704	1.745052
O	0.000000	-1.958857	1.744547
Cu	0.000000	0.000000	1.609749
C	4.195881	0.561684	1.553295
H	4.375391	-0.167849	0.747440
H	4.472298	0.059920	2.494259
H	4.841605	1.437833	1.404658
C	2.731974	0.901067	1.591260
C	2.343389	2.247359	1.469966
H	3.126802	2.998662	1.342055
C	1.014354	2.692070	1.590216
C	0.736309	4.168830	1.550828
H	0.249229	4.467382	2.492774
H	0.012572	4.377373	0.746753
H	1.638247	4.777215	1.398908
C	-0.790189	0.821854	-1.335491
H	-1.427144	1.484913	-0.734700

C	-0.832306	0.865234	-2.719226
H	-1.508980	1.568660	-3.207007
C	-0.867686	0.900818	-5.550651
H	-0.650086	1.960501	-5.322359
H	-1.934880	0.722031	-5.323565
H	-0.730650	0.759731	-6.630418
O	1.956825	-0.081704	1.745052
O	0.000000	1.958857	1.744547

Table S18. Atomic coordinates for the optimized structure Cu(acac)2-hmta

MO6 DEF2SVP

C	3.567270	1.357122	2.463975
C	2.751287	1.297999	1.204795
C	3.411517	1.246569	-0.035927
H	4.503836	1.275572	-0.039944
C	2.744733	1.189009	-1.272767
C	3.554466	1.161499	-2.537435
C	1.040338	-1.726294	0.689665
H	1.961177	-1.447231	0.144844
H	1.119078	-1.326352	1.716313
C	-0.212625	-1.624299	-1.340356
H	-1.071719	-1.145720	-1.841956
H	0.697057	-1.337949	-1.896473
O	1.500368	1.284014	1.375114
O	1.492859	1.149966	-1.433765
Cu	0.127201	1.195243	-0.022369
N	-0.110973	-1.088771	0.028655
N	0.902390	-3.173386	0.722656
C	-3.246627	1.809772	-2.533436

C	-2.442907	1.699854	-1.269499
C	-3.081219	1.903626	-0.033291
H	-4.146775	2.145714	-0.036899
C	-2.417960	1.845581	1.205770
C	-3.200430	2.087987	2.464264
C	0.778960	-3.657851	-0.650376
C	-0.328510	-3.502624	1.437511
H	0.672657	-4.757123	-0.638892
H	-0.254979	-3.135427	2.476417
H	1.699649	-3.409421	-1.207488
H	-0.447202	-4.600177	1.470135
C	-1.328376	-1.467977	0.765379
C	-1.573410	-3.401340	-0.574552
H	-2.199537	-0.994123	0.276115
H	-2.452413	-2.956738	-1.074019
H	-1.255416	-1.066123	1.791761
H	-1.705591	-4.497793	-0.562318
O	-1.222700	1.417315	-1.431077
O	-1.193924	1.587357	1.374789
N	-0.370178	-3.070111	-1.333593
N	-1.502500	-2.911177	0.800178
H	4.648065	1.417456	2.278399
H	3.353220	0.461445	3.070043
H	3.245197	2.222737	3.063519
H	4.637751	1.194900	-2.359640
H	3.260070	2.013329	-3.170574
H	3.305242	0.251472	-3.106646
H	-4.301624	2.058729	-2.356725
H	-3.183200	0.857910	-3.085336
H	-2.792291	2.574941	-3.182455

H	-4.249597	2.354392	2.278583
H	-2.712811	2.886301	3.045155
H	-3.161679	1.180349	3.088713

Table S19. Atomic coordinates for the optimized structure Cu(acac)2-dabco

MO6 DEF2SVP

C	2.604702	-1.197337	1.281728
Cu	0.000278	-0.937392	0.029063
N	-0.000305	1.367872	-0.051767
O	1.364236	-1.026948	1.440941
C	3.413844	-1.230614	2.547040
N	-0.001150	3.897852	-0.128465
O	1.353411	-1.221791	-1.363121
C	3.261166	-1.347741	0.047156
H	4.344574	-1.490454	0.052733
C	2.595627	-1.361601	-1.191850
C	3.397876	-1.542209	-2.448811
H	2.995104	-2.396707	-3.014771
H	4.469306	-1.695877	-2.262255
H	3.263513	-0.654551	-3.088496
C	1.194777	1.841127	-0.758523
H	1.192123	1.388527	-1.765375
H	2.082334	1.443749	-0.234216
C	1.186003	3.388835	-0.800055
H	2.077460	3.811488	-0.304508
H	1.189706	3.765225	-1.837983
C	-0.000615	1.902409	1.314992
H	0.881408	1.494271	1.837053
H	-0.882494	1.493697	1.836864
C	-0.001037	3.449037	1.257699
H	-0.887132	3.872963	1.761844
H	0.884857	3.873416	1.761821
C	-1.195592	1.840331	-0.758772
H	-2.083029	1.442245	-0.234820

H	-1.192267	1.387881	-1.765690
C	-1.187938	3.388059	-0.800104
H	-1.191961	3.764584	-1.837982
H	-2.079684	3.809988	-0.304459
O	-1.352719	-1.222617	-1.363123
C	-2.594871	-1.362992	-1.191826
C	-3.397083	-1.543705	-2.448800
C	-3.260397	-1.349593	0.047190
H	-4.343737	-1.492814	0.052757
C	-2.604019	-1.198679	1.281745
O	-1.363622	-1.027783	1.440917
C	-3.413213	-1.231449	2.547032
H	-3.255517	-0.291769	3.100627
H	-4.487838	-1.375602	2.371571
H	-3.035723	-2.039311	3.193662
H	4.488842	-1.371720	2.371398
H	3.038456	-2.040968	3.191806
H	3.253561	-0.292644	3.102739
H	-4.468365	-1.698363	-2.262226
H	-2.993615	-2.397532	-3.015267
H	-3.263570	-0.655571	-3.088011

Table S20. Atomic coordinates for the optimized structure Cu(acac)2-dpe

MO6 DEF2SVP

C	-2.751208	-2.594694	1.315172
C	-2.746998	-3.245337	0.068445
H	-2.618365	-4.330550	0.056119
C	-2.985965	-2.594498	-1.155137
C	-3.066813	-3.415318	-2.411163
C	-2.593086	-3.415123	2.564090
C	0.148844	0.000234	0.939935
H	-0.368399	-0.000163	1.908147
C	1.533721	0.000429	0.859207
H	2.125905	0.000285	1.777968
C	2.148252	0.000870	-0.403391
C	1.303012	0.001146	-1.522272
H	1.726520	0.001483	-2.531249
C	-0.075328	0.000936	-1.342128
H	-0.767391	0.001140	-2.194361
C	3.595191	0.001046	-0.600279
H	3.915524	0.001948	-1.649900
N	-0.637999	0.000486	-0.136734
O	-2.889749	-1.354298	1.501048
O	-3.156740	-1.354049	-1.311812
Cu	-2.913215	-0.000318	0.084438
C	-2.987120	2.594501	-1.153689
C	-2.748618	3.244772	0.070278
H	-2.620371	4.330033	0.058549
C	-2.752746	2.593449	1.316658
C	-2.595056	3.413265	2.566028
H	-1.738660	3.030224	3.143566
H	-2.453918	4.484346	2.367782

H	-3.484849	3.274219	3.200530
C	-3.068134	3.415985	-2.409278
C	7.981587	0.002592	-1.161880
H	8.471686	0.004629	-2.144538
C	6.593931	0.002783	-1.088247
H	6.004545	0.005035	-2.009283
C	5.976440	0.000198	0.171605
C	6.824136	-0.002360	1.287037
H	6.404167	-0.004410	2.297936
C	8.204106	-0.002315	1.099061
H	8.872176	-0.004353	1.970669
C	4.529087	0.000065	0.367483
H	4.208707	-0.001105	1.417253
N	8.782248	0.000097	-0.097165
O	-3.157429	1.354077	-1.311080
O	-2.890801	1.352894	1.501828
H	-2.885899	-4.485548	-2.242857
H	-2.338751	-3.029629	-3.142428
H	-4.062377	-3.281030	-2.863396
H	-2.450836	-4.485937	2.365202
H	-3.483208	-3.277369	3.198414
H	-1.737242	-3.031599	3.142118
H	-2.887444	4.486164	-2.240401
H	-4.063674	3.281754	-2.861585
H	-2.339997	3.030824	-3.140744

Table S21. Atomic coordinates for the optimized structure $Cu(acac)_2(bipy)$

MO6 DEF2SVP

C	-4.195881	-0.561684	1.553295
H	-4.375391	0.167849	0.747440
H	-4.472298	-0.059920	2.494259
H	-4.841605	-1.437833	1.404658
C	-2.731974	-0.901067	1.591260
C	-2.343389	-2.247359	1.469966
H	-3.126802	-2.998662	1.342055
C	-1.014354	-2.692070	1.590216
C	-0.736309	-4.168830	1.550828
H	-0.249229	-4.467382	2.492774
H	-0.012572	-4.377373	0.746753
H	-1.638247	-4.777215	1.398908
C	0.790189	-0.821854	-1.335491
H	1.427144	-1.484913	-0.734700
C	0.832306	-0.865234	-2.719226
H	1.508980	-1.568660	-3.207007
C	0.000000	0.000000	-3.465468
C	0.867686	-0.900818	-5.550651
H	0.650086	-1.960501	-5.322359
H	1.934880	-0.722031	-5.323565
H	0.730650	-0.759731	-6.630418
N	0.000000	0.000000	-0.644814
N	0.000000	0.000000	-4.832825
O	-1.956825	0.081704	1.745052
O	0.000000	-1.958857	1.744547
Cu	0.000000	0.000000	1.609749
C	4.195881	0.561684	1.553295

H	4.375391	-0.167849	0.747440
H	4.472298	0.059920	2.494259
H	4.841605	1.437833	1.404658
C	2.731974	0.901067	1.591260
C	2.343389	2.247359	1.469966
H	3.126802	2.998662	1.342055
C	1.014354	2.692070	1.590216
C	0.736309	4.168830	1.550828
H	0.249229	4.467382	2.492774
H	0.012572	4.377373	0.746753
H	1.638247	4.777215	1.398908
C	-0.790189	0.821854	-1.335491
H	-1.427144	1.484913	-0.734700
C	-0.832306	0.865234	-2.719226
H	-1.508980	1.568660	-3.207007
C	-0.867686	0.900818	-5.550651
H	-0.650086	1.960501	-5.322359
H	-1.934880	0.722031	-5.323565
H	-0.730650	0.759731	-6.630418
O	1.956825	-0.081704	1.745052
O	0.000000	1.958857	1.744547

Table S22. Atomic coordinates for the optimized structure Cu(acac)2 1,2-DITFB

M062X def2TZVPD

F	-2.068238	3.023142	-0.002547
C	-2.959392	2.035241	-0.001926
C	-2.557175	0.708781	-0.000673
I	-0.508995	0.330164	0.000230
I	-3.110026	-2.318814	0.001829
C	-3.539719	-0.284310	-0.000003
F	-5.839514	-0.842234	0.000055
C	-4.878313	0.074134	-0.000597
C	-5.263746	1.404154	-0.001886
F	-6.547192	1.730467	-0.002459
F	-4.653704	3.666465	-0.003736
C	-4.297614	2.390955	-0.002543
Cu	3.763083	-0.006387	-0.000113
O	2.497300	1.455884	0.002173
C	1.554934	3.602831	0.004436
H	0.946644	3.385754	-0.874943
H	1.830807	4.654074	0.012091
H	0.938987	3.374162	0.875410
C	2.754459	2.698283	0.003284
C	4.038411	3.248156	0.002829
H	4.131499	4.322681	0.003916
O	5.262686	1.219621	-0.000348
C	6.545853	3.176683	-0.000254
H	7.105296	2.857935	-0.880457
H	6.451709	4.259422	0.002683
H	7.109328	2.853301	0.875647
C	5.211503	2.483052	0.000845

O	2.230815	-1.203357	0.000416
C	0.908222	-3.142684	0.000548
H	0.346176	-2.810453	0.875225
H	0.988370	-4.226713	0.002452
H	0.347422	-2.813725	-0.876204
C	2.253690	-2.473254	0.000010
C	3.410527	-3.254167	-0.001150
H	3.298209	-4.326832	-0.001260
O	4.998879	-1.497273	-0.002425
C	5.886116	-3.662567	-0.004564
H	6.501917	-3.452091	0.870496
H	5.587418	-4.707503	-0.001119
H	6.495099	-3.456320	-0.885453
C	4.708945	-2.726885	-0.002462

Table S23. Atomic coordinates for the optimized structure Cu(acac)$_2$·1,3-DITFB

M062X def2TZVPD

F	1.740702	3.166310	-0.000081
C	2.565802	2.127092	-0.000207
C	2.071451	0.833084	0.000592
I	0.025712	0.472905	0.001932
F	2.543083	-1.457806	0.001168
C	2.990584	-0.205240	0.000423
I	5.701109	-1.566376	-0.001089
C	4.360403	0.011307	-0.000577
C	4.815151	1.319984	-0.001342
F	6.113039	1.588409	-0.002286
F	4.376505	3.628857	-0.001958
C	3.926479	2.380511	-0.001185
Cu	-4.175832	-0.323981	-0.000085
O	-3.042513	1.250973	0.001751
C	-2.311405	3.482084	0.002571
H	-1.681308	3.321793	0.878709
H	-2.690547	4.500619	0.002829
H	-1.680337	3.322700	-0.873054
C	-3.416934	2.464413	0.001372
C	-4.746050	2.891804	-0.000071
H	-4.937625	3.953177	-0.000026
O	-5.779988	0.760793	-0.002359
C	-7.236886	2.592104	-0.003579
H	-7.768180	2.219934	0.873064
H	-7.241998	3.678905	-0.002365
H	-7.765106	2.222012	-0.882980
C	-5.844975	2.022892	-0.001867

O	-2.548675	-1.378407	0.002622
C	-1.053450	-3.187744	0.005599
H	-0.523116	-2.808116	-0.869034
H	-1.033766	-4.274394	0.005875
H	-0.526704	-2.807748	0.882241
C	-2.454391	-2.644565	0.002681
C	-3.536419	-3.527150	0.000269
H	-3.328227	-4.585399	0.000402
O	-5.275149	-1.918601	-0.002258
C	-5.965366	-4.154666	-0.004575
H	-6.591968	-4.001808	-0.883958
H	-5.574443	-5.168738	-0.003864
H	-6.595825	-4.002023	0.872093
C	-4.876095	-3.117832	-0.002070

Table S24. Atomic coordinates for the optimized structure Cu(acac)2 ·1,4-DITFB

M062X def2TZVPD

F	-0.000031	2.355786	2.014505
C	-0.000007	1.185611	2.646094
C	0.000000	0.000000	1.929843
I	0.000000	0.000000	-0.145169
F	0.000031	-2.355786	2.014505
C	0.000007	-1.185611	2.646094
F	-0.000007	-2.355700	4.664841
C	0.000000	-1.188876	4.030692
C	0.000000	0.000000	4.741703
I	0.000000	0.000000	6.809354
F	0.000007	2.355700	4.664841
C	0.000000	1.188876	4.030692
Cu	0.000000	0.000000	-4.419118
O	-0.001666	1.337159	-3.014035
C	-0.004717	3.393094	-1.880759
H	-0.880327	3.117351	-1.291154
H	-0.005912	4.464434	-2.063248
H	0.871207	3.119367	-1.290688
C	-0.003426	2.599073	-3.156397
C	-0.004142	3.265607	-4.383147
H	-0.005628	4.344110	-4.374544
O	-0.001053	1.363277	-5.794410
C	-0.003694	3.433010	-6.886354
H	-0.881544	3.165683	-7.475596
H	-0.004674	4.501906	-6.689932
H	0.874533	3.167294	-7.475759
C	-0.002856	2.615588	-5.624125

O	0.001666	-1.337159	-3.014035
C	0.004717	-3.393094	-1.880759
H	0.880327	-3.117351	-1.291154
H	0.005912	-4.464434	-2.063248
H	-0.871207	-3.119367	-1.290688
C	0.003426	-2.599073	-3.156397
C	0.004142	-3.265607	-4.383147
H	0.005628	-4.344110	-4.374544
O	0.001053	-1.363277	-5.794410
C	0.003694	-3.433010	-6.886354
H	0.881544	-3.165683	-7.475596
H	0.004674	-4.501906	-6.689932
H	-0.874533	-3.167294	-7.475759
C	0.002856	-2.615588	-5.624125

Table S25. Atomic coordinates for the optimized structure $VO(acac)_2$·1,4-DITFB

M062X def2TZVPD

I	0.134317	0.017228	-0.188584
F	-2.028363	2.365607	-0.059839
F	-2.013518	-2.345771	-0.109072
C	-2.655446	1.193439	-0.037597
C	-2.648017	-1.178208	-0.062477
C	-1.936870	0.010218	-0.085702
I	-6.809168	-0.008187	0.162821
F	-4.660287	-2.355230	0.026701
F	-4.675073	2.355640	0.076725
C	-4.030738	-1.186304	0.007899
C	-4.038221	1.191342	0.033054
C	-4.744545	-0.000088	0.057063
V	4.261681	-0.004606	0.548536
O	3.033820	1.312965	-0.294882
O	3.975780	0.000143	2.074766
C	4.399325	3.235118	-0.298615
H	4.427415	4.299783	-0.467069
C	1.955822	3.320551	-0.861956
H	1.538377	2.862800	-1.759698
H	2.159814	4.372668	-1.041514
H	1.205057	3.218368	-0.076078
C	3.186184	2.566513	-0.456264
C	5.596225	2.569048	-0.019098
O	5.693624	1.322940	0.191932
C	6.888354	3.329962	0.023715
H	7.332782	3.204692	1.011906
H	6.754169	4.387198	-0.188356

H	7.577301	2.891156	-0.698841
O	5.681228	-1.347812	0.200191
C	4.368336	-3.249737	-0.281027
H	4.385732	-4.315637	-0.443022
C	6.855758	-3.367896	0.045945
H	7.549680	-2.941385	-0.679213
H	6.710887	-4.425186	-0.158717
H	7.300708	-3.240106	1.033569
C	5.571497	-2.594147	-0.003457
C	3.162667	-2.569340	-0.445291
O	3.023528	-1.313359	-0.291421
C	1.924995	-3.312084	-0.849490
H	1.173374	-3.196364	-0.066339
H	2.117552	-4.367535	-1.021974
H	1.514985	-2.855186	-1.751099

Table S26. Atomic coordinates for the optimized structure $VO(acac)_2py$

MO6 DEF2SVP

V	0.000000	0.000000	1.268445
O	0.000000	0.000000	2.824431
O	0.663312	1.846612	0.868366
O	-1.850319	0.650668	0.865168
N	0.000000	0.000000	-1.235882
C	0.000000	2.882408	0.572125
C	-1.394528	2.933143	0.412691
H	-1.850219	3.891794	0.153833
C	-2.235068	1.818543	0.567845
C	-3.710277	1.970559	0.352276
H	-4.019065	1.311453	-0.475882
H	-4.012729	3.002001	0.127546
H	-4.243394	1.615002	1.247939
C	0.812487	4.124249	0.363352
H	1.417921	4.313536	1.263639
H	0.203262	5.009011	0.135538
H	1.524996	3.950236	-0.459854
C	-1.031195	-0.504002	-1.913651
H	-1.851923	-0.904938	-1.304567
C	-1.076909	-0.526360	-3.305139
H	-1.942608	-0.949518	-3.820548
C	0.000000	0.000000	-4.012905
H	0.000000	0.000000	-5.106889
C	1.031195	0.504002	-1.913651
H	1.851923	0.904938	-1.304567
C	1.076909	0.526360	-3.305139
H	1.942608	0.949518	-3.820548

O	-0.663312	-1.846612	0.868366
O	1.850319	-0.650668	0.865168
C	0.000000	-2.882408	0.572125
C	1.394528	-2.933143	0.412691
H	1.850219	-3.891794	0.153833
C	2.235068	-1.818543	0.567845
C	3.710277	-1.970559	0.352276
H	4.019065	-1.311453	-0.475882
H	4.012729	-3.002001	0.127546
H	4.243394	-1.615002	1.247939
C	-0.812487	-4.124249	0.363352
H	-1.417921	-4.313536	1.263639
H	-0.203262	-5.009011	0.135538
H	-1.524996	-3.950236	-0.459854

Table S27. Atomic coordinates for the optimized structure $VO(acac)_2$dmap

V	0.868548	-0.005698	-0.956370
O	1.050380	0.107395	1.237710
N	-5.324769	-0.256614	0.285334
C	-2.092824	0.540537	-1.270868
H	-1.666510	1.084151	-2.120430
C	-3.457337	0.520642	-1.058768
H	-4.101830	1.061398	-1.752906
C	-3.984381	-0.198917	0.038187
C	-3.042414	-0.854428	0.863279
H	-3.350188	-1.431399	1.736349
C	-1.695162	-0.770040	0.567379
H	-0.947599	-1.259122	1.199309
C	-6.246686	0.429852	-0.587607
H	-6.071712	1.521095	-0.602574
H	-7.274021	0.263029	-0.239880
H	-6.182521	0.064764	-1.628750
C	-5.815535	-1.007158	1.415911
H	-5.543826	-2.076726	1.354152
H	-6.910564	-0.944450	1.450072
H	-5.428433	-0.616620	2.374749
C	2.041464	-2.576614	-0.093078
C	3.337741	-2.045408	0.002342
H	4.135908	-2.695047	0.369438
C	5.089337	-0.266141	-0.261441
H	5.442036	0.094247	-1.240589
H	5.773719	-1.037301	0.116907
H	5.105147	0.604790	0.414808
C	1.803750	-4.005429	0.294921

H	1.066559	-4.037713	1.113288
H	2.717786	-4.527060	0.608875
H	1.350546	-4.538366	-0.555873
C	1.952634	0.753161	3.307919
H	2.733159	-0.025587	3.273102
H	1.103672	0.317431	3.858479
H	2.334850	1.626266	3.854788
C	1.529177	1.062145	1.898800
C	1.688486	2.374485	1.405735
H	2.086262	3.142368	2.074200
C	1.274667	2.758382	0.122847
C	1.351504	4.202331	-0.277512
H	2.004927	4.295328	-1.159831
H	1.724712	4.854471	0.523918
H	0.352751	4.544315	-0.592557
O	0.618550	-0.103055	-2.497791
O	1.017810	-1.948263	-0.490711
O	2.862219	0.113737	-0.852684
O	0.813556	1.979472	-0.763710
N	-1.214471	-0.089449	-0.480032
**C**	**3.672767**	**-0.738243**	**-0.390073**

Table S27. Atomic coordinates for the optimized structure dmap·1,4-DITFB

M062X def2TZVPD

I	1.064130	0.001990	-0.006981
F	-1.119471	2.354873	0.089643
F	-1.117525	-2.353200	-0.096976
C	-1.027511	0.000865	-0.003821
C	-1.747233	1.183207	0.044258
C	-1.746260	-1.182135	-0.049501
I	-5.910825	-0.001506	0.004984
F	-3.765630	-2.354373	-0.092882
F	-3.767567	2.353516	0.094618
C	-3.842760	-0.000470	0.001004
C	-3.131074	-1.187634	-0.047427
C	-3.132048	1.187386	0.046923
N	3.912115	0.002510	-0.006633
N	8.094689	-0.002247	0.008865
C	4.616846	0.130824	-1.129595
C	5.995125	0.136206	-1.186536
C	6.734306	-0.000708	0.003851
C	5.986021	-0.135948	1.188715
C	4.608220	-0.127419	1.121516
C	8.810285	-0.145710	1.258457
C	8.819785	0.138849	-1.235509
H	4.041586	0.236215	-2.043617
H	6.480830	0.245522	-2.144035
H	6.464342	-0.246425	2.149787
H	4.025915	-0.231548	2.031229
H	9.878266	-0.124528	1.061810
H	8.571837	-1.093549	1.749312

H	8.573952	0.667741	1.950289
H	8.586611	-0.674388	-1.928678
H	8.587187	1.087005	-1.728534
H	9.886236	0.115266	-1.030991

Table S28. Atomic coordinates for the optimized structure dpe·1,2-DITFB

M062X def2TZVPD

I	-3.421221	2.408995	-0.176767
I	-1.188292	-0.568554	-0.052395
F	-6.316525	1.328147	0.072809
F	-7.362871	-1.113419	0.320926
F	-3.105787	-3.010063	0.237062
F	-5.749957	-3.287566	0.402070
C	-4.115496	0.457886	0.020151
C	-5.488250	0.290166	0.108665
C	-3.276114	-0.658018	0.062545
C	-3.853604	-1.911107	0.191802
C	-6.048727	-0.969340	0.237867
C	-5.225677	-2.077385	0.279234
N	1.729299	-0.490161	-0.195489
N	11.093425	0.173844	0.214534
C	5.992074	-0.575915	-0.328211
H	6.398722	-1.451135	-0.824248
C	4.528212	-0.511134	-0.280907
C	8.286263	0.247169	0.185660
C	3.819610	0.639660	0.068657
H	4.328963	1.565333	0.298380
C	3.779705	-1.642592	-0.600146
H	4.271901	-2.563141	-0.886751
C	6.821752	0.315153	0.216759

H	6.413074	1.157302	0.766177
C	2.437861	0.598778	0.099033
H	1.867246	1.481252	0.366871
C	2.396647	-1.586342	-0.539920
H	1.796916	-2.458052	-0.778428
C	9.000678	-0.602733	-0.658944
H	8.496845	-1.247555	-1.366104
C	9.029496	1.066155	1.032212
H	8.533420	1.753254	1.706233
C	10.383772	-0.601663	-0.603387
H	10.953360	-1.255308	-1.254779
C	10.414591	0.990482	1.012141
H	11.004228	1.619691	1.669866

Table S29. Atomic coordinates for the optimized structure dpe·1,3-DITFB

M062X def2TZVPD

I	6.292029	-1.694462	-0.057210
I	0.616998	0.374358	0.059201
F	2.359308	3.059047	0.024347
F	3.136106	-1.568560	0.006197
F	6.721388	1.457730	-0.066943
F	4.995805	3.507533	-0.030734
C	3.177421	2.014457	0.006711
C	5.422047	1.196367	-0.040223
C	4.539167	2.261250	-0.021692
C	3.588975	-0.318161	-0.002663
C	4.959945	-0.109552	-0.030929
C	2.674789	0.724003	0.016643
N	-2.258785	-0.085001	0.104676
C	-5.033464	-0.462987	0.101142
C	-4.152262	-1.519930	-0.120156
H	-4.528243	-2.518374	-0.303357
C	-3.094600	0.928000	0.328565
H	-2.635470	1.893243	0.512284
C	-2.786898	-1.284768	-0.112570
H	-2.085826	-2.093518	-0.287623
C	-4.470435	0.791820	0.340230
H	-5.087472	1.654427	0.550448
C	-6.478257	-0.710359	0.076944
H	-6.765916	-1.756546	0.087127
N	-11.647900	-0.385605	-0.163590
C	-8.867278	-0.013135	-0.036440
C	-9.726979	1.021570	-0.396944

H	-9.334960	2.000968	-0.641060
C	-10.827426	-1.372694	0.192107
H	-11.296815	-2.320630	0.431004
C	-11.093339	0.787021	-0.448737
H	-11.772470	1.583534	-0.732442
C	-9.451857	-1.241479	0.272863
H	-8.854569	-2.085533	0.589831
C	-7.421713	0.229815	0.003645
H	-7.130355	1.273016	-0.066129

Table S30. Atomic coordinates for the optimized structure dpe·1,4-DITFB

M062X def2TZVPD

I	7.343494	0.223897	-0.159358
F	5.335921	-2.245396	-0.287876
F	5.076319	2.434995	0.179292
C	5.282222	0.099409	-0.058483
C	4.638704	-1.124176	-0.143394
C	4.507688	1.237776	0.092360
C	3.257651	-1.203064	-0.080509
F	2.693509	-2.403885	-0.168623
C	2.476961	-0.068962	0.068628
I	0.397036	-0.192579	0.153855
C	3.127109	1.150900	0.154432
F	2.433867	2.276410	0.298446
N	-2.507159	-0.343758	0.228099
C	-6.766965	-0.539659	0.125496
C	-5.305230	-0.438573	0.173536
C	-4.620950	0.769048	0.322333
C	-3.238485	0.764158	0.340309
C	-3.151530	-1.498557	0.097855
C	-4.533370	-1.594478	0.069682
H	-7.161910	-1.545888	0.219650
H	-5.150075	1.704536	0.440167
H	-2.686260	1.690549	0.454775
H	-2.534112	-2.386102	0.012416
H	-5.006415	-2.561986	-0.039531
C	-7.605613	0.480543	-0.062359
C	-9.067271	0.382559	-0.127694
C	-9.783197	-0.746171	0.271798

C	-11.162697	-0.744579	0.157762
N	-11.867714	0.282935	-0.312471
C	-11.187957	1.361835	-0.683754
C	-9.806273	1.460868	-0.608286
H	-9.309108	2.368554	-0.926752
H	-11.773980	2.193001	-1.060480
H	-11.733333	-1.613843	0.465565
H	-9.284342	-1.613069	0.683254
H	-7.205762	1.477786	-0.216244

Table S31. Atomic coordinates for the optimized structure dpe·1,4-Br2TFB

M062X def2TZVPD

C	-6.984711	0.404671	-0.088434
H	-6.777107	1.424999	-0.394662
C	-8.405569	0.043188	-0.058378
C	-9.342962	0.891487	-0.642964
H	-9.029251	1.815067	-1.113189
C	-10.685145	0.541218	-0.625936
H	-11.423508	1.191671	-1.081970
N	-11.146105	-0.573186	-0.069365
C	-10.251747	-1.379603	0.499731
H	-10.645077	-2.280240	0.958193
C	-8.892260	-1.121864	0.535045
H	-8.231540	-1.816565	1.035568
C	-5.969982	-0.416452	0.186181
H	-6.174611	-1.452879	0.434337
C	-4.549131	-0.057062	0.143451
C	-3.585297	-1.062944	0.164828
H	-3.879261	-2.103582	0.217006
C	-2.242301	-0.723465	0.111575
H	-1.478777	-1.494411	0.123091
N	-1.808690	0.530634	0.046361
C	-2.726318	1.496347	0.036588
H	-2.350483	2.512433	-0.006885
C	-4.088135	1.259105	0.085621
H	-4.773047	2.095822	0.095685
C	5.768272	-0.177182	-0.025002
C	5.224245	1.091644	0.095753
C	4.909574	-1.259245	-0.135831

Br	7.620411	-0.423475	-0.037577
F	6.010860	2.152896	0.204805
F	5.389424	-2.489451	-0.252951
C	3.851840	1.272319	0.104769
C	2.989030	0.193090	-0.005928
C	3.537866	-1.074074	-0.126017
F	2.753862	-2.138824	-0.234319
Br	1.132008	0.441140	0.006367
F	3.375624	2.504293	0.222010

Table S332. Atomic coordinates for the optimized structure dpe·1,4-I2Ph

M062X def2TZVPD

I	-0.978984	-0.348189	-0.185793
C	-3.061698	-0.164874	-0.073223
C	-3.677658	-0.001536	1.160389
C	-3.827166	-0.206858	-1.230838
H	-3.086037	0.031288	2.065491
H	-3.352245	-0.334228	-2.194408
C	-5.058463	0.120539	1.238809
C	-5.815999	0.077873	0.078147
I	-7.894529	0.261155	0.194470
C	-5.208496	-0.085466	-1.157644
H	-5.801938	-0.118908	-2.061050
H	-5.535467	0.247537	2.200870
N	2.079352	-0.362004	-0.282127
C	2.977929	-1.341158	-0.371471
C	4.344653	-1.130224	-0.339923
C	4.830327	0.171041	-0.202532
C	3.886160	1.192648	-0.122370
C	2.536394	0.879573	-0.161933
C	6.258081	0.499679	-0.144245
H	2.581808	-2.344736	-0.479233
H	5.015248	-1.972619	-0.440021
H	4.200613	2.223820	-0.023002
H	1.786175	1.660670	-0.094624
H	6.492850	1.553030	-0.258298
C	7.243762	-0.372162	0.074054
C	8.671082	-0.043545	0.146776
C	9.206044	1.168360	-0.290790

C	10.567046	1.388498	-0.167553
N	11.419115	0.503158	0.346446
C	10.912391	-0.654924	0.754271
C	9.564611	-0.973287	0.673193
H	9.212478	-1.935699	1.022877
H	11.616784	-1.368961	1.166829
H	10.997663	2.324844	-0.505027
H	8.582026	1.929421	-0.739229
H	7.002973	-1.415772	0.249975

Table S33. Atomic coordinates for the optimized structure bipy ·1,4-DITFB

M062X def2TZVPD

N	-3.270505	0.022825	-0.019088
C	-3.948691	-1.117564	-0.110147
H	-3.356166	-2.021819	-0.196395
C	-5.332590	-1.179080	-0.103432
H	-5.832795	-2.133523	-0.199796
C	-6.062783	0.001817	-0.001547
C	-5.349194	1.193588	0.091021
H	-5.862426	2.140416	0.193842
C	-3.964542	1.152916	0.080452
H	-3.384731	2.065972	0.159733
C	-7.542222	-0.009407	0.008337
C	-8.276308	0.985535	-0.629634
C	-9.661530	0.927373	-0.593350
N	-10.342424	-0.030894	0.027446
C	-9.638528	-0.978524	0.638845
C	-8.252218	-1.015406	0.656217
H	-7.737066	-1.804135	1.188777
H	-10.209507	-1.748441	1.145938
H	-10.251091	1.688462	-1.092423
H	-7.780615	1.782153	-1.168893
C	2.454229	1.186453	0.089067
F	1.829442	2.356582	0.180052
C	1.731321	0.009171	-0.008674
I	-0.353633	0.019700	-0.015910
C	3.838951	1.183253	0.095391
F	4.478887	2.343080	0.191783
C	2.442920	-1.175461	-0.100564

C	3.827590	-1.186462	-0.095099
F	4.456371	-2.352830	-0.185980
C	4.544372	-0.005270	0.003353
I	6.612038	-0.016292	0.013787
F	1.806944	-2.339145	-0.196761

Table S34. Atomic coordinates for the optimized structure hmta·1,4-DITFB

C	6.505982	0.173854	1.296880
H	6.187353	0.308093	2.332228
H	7.596693	0.172017	1.261286
N	6.006226	1.295133	0.494413
C	6.426491	1.085449	-0.894886
H	7.516916	1.086797	-0.938375
N	5.930841	-0.184162	-1.436498
C	6.432542	-1.269843	-0.587603
H	7.522986	-1.276774	-0.630083
H	6.058835	-2.221503	-0.969940
N	6.012518	-1.118397	0.809319
C	4.551744	-1.092627	0.846835
H	4.162000	-2.040070	0.468859
H	4.217835	-0.961268	1.878145
C	4.545655	1.272702	0.538419
H	4.211828	1.408665	1.569147
H	4.151216	2.089578	-0.069521
N	4.003465	0.002272	0.030244
C	4.471938	-0.176955	-1.353605
H	6.048141	1.905275	-1.508273
H	4.077914	0.637852	-1.964692
H	4.081970	-1.123007	-1.735000
C	-1.659389	-1.183397	0.021066
F	-1.027779	-2.353850	0.027814
C	-0.943361	0.001470	0.028977
I	1.149635	0.002792	0.047828
C	-3.044118	-1.188769	0.005426

F	-3.677618	-2.356074	-0.002017
C	-1.660929	1.185390	0.020980
C	-3.045671	1.188952	0.005349
F	-3.680692	2.355425	-0.002180
C	-3.755752	-0.000370	-0.002866
I	-5.823287	-0.001737	-0.028723
F	-1.030831	2.356653	0.027656

Table S35. Atomic coordinates for the optimized structure dabco·1,4-DITFB

M062X def2TZVPD

I	1.547160	-0.002055	0.001744
I	-5.433438	0.001251	-0.001026
F	-0.640797	-2.356587	0.001002
F	-3.290257	-2.355658	-0.000082
F	-3.287993	2.356108	-0.000078
F	-0.638531	2.354468	0.001013
C	-0.551417	-0.001082	0.001078
C	-1.270136	-1.184214	0.000784
C	-2.654974	-1.188771	0.000208
C	-3.365478	0.000257	-0.000108
C	-2.653851	1.188595	0.000219
C	-1.269013	1.182709	0.000800
N	4.330410	-0.000579	0.000886
N	6.876243	0.002993	-0.003236
C	4.823006	-0.382208	-1.327358
H	4.386050	0.298867	-2.060000
H	4.452704	-1.385686	-1.545420
C	6.376938	-0.324937	-1.338780
H	6.805418	-1.281379	-1.641792
H	6.738825	0.435748	-2.032289
C	4.823235	1.340963	0.332819
H	4.389361	1.634768	1.290357
H	4.449630	2.031496	-0.425574
C	6.377306	1.323175	0.382375
H	6.802872	2.064032	-0.296022
H	6.742929	1.543665	1.386504
C	4.827524	-0.958465	0.994905
H	4.393385	-1.934938	0.772443
H	4.457853	-0.646634	1.973568
C	6.381579	-0.991594	0.948961
H	6.810635	-0.774556	1.928274
H	6.746401	-1.971211	0.636219

References.

(1) Stilinović, V.; Grgurić, T.; Piteša, T.; Nemec, V.; Cinčić, D. Bifurcated and Monocentric Halogen Bonds in Cocrystals of Metal(II) Acetylacetonates with p-Dihalotetrafluorobenzenes. *Crystal Growth & Design* **2018**, *19* (2), 1245-1256. DOI: 10.1021/acs.cgd.8b01659.
(2) Spackman, P. R.; Turner, M. J.; McKinnon, J. J.; Wolff, S. K.; Grimwood, D. J.; Jayatilaka, D.; Spackman, M. A. CrystalExplorer: a program for Hirshfeld surface analysis, visualization and quantitative analysis of molecular crystals. *Journal of Applied Crystallography* **2021**, *54* (3), 1006-1011. DOI: 10.1107/s1600576721002910.
(3) *Gaussian 16 Rev. C.01*; Wallingford, CT, 2016. (accessed.
(4) Zhao, Y.; Truhlar, D. G. The M06 suite of density functionals for main group thermochemistry, thermochemical kinetics, noncovalent interactions, excited states, and transition elements: two new functionals and systematic testing of four M06-class functionals and 12 other functionals. *Theoretical Chemistry Accounts* **2007**, *120* (1-3), 215-241. DOI: 10.1007/s00214-007-0310-x.
(5) Weigend, F.; Ahlrichs, R. Balanced basis sets of split valence, triple zeta valence and quadruple zeta valence quality for H to Rn: Design and assessment of accuracy. *Physical Chemistry Chemical Physics* **2005**, *7* (18), 3297. DOI: 10.1039/b508541a.
(6) Zhao, Y.; Truhlar, D. G. The M06 suite of density functionals for main group thermochemistry, thermochemical kinetics, noncovalent interactions, excited states, and transition elements: two new functionals and systematic testing of four M06-class functionals and 12 other functionals. *Theoretical Chemistry Accounts* **2008**, *120* (1), 215-241. DOI: 10.1007/s00214-007-0310-x.
(7) Aakeröy, C. B.; Wijethunga, T. K.; Desper, J. Practical crystal engineering using halogen bonding: A hierarchy based on calculated molecular electrostatic potential surfaces. *Journal of Molecular Structure* **2014**, *1072*, 20-27. DOI: 10.1016/j.molstruc.2014.02.022.
(8) Korobeynikov, N. A.; Usoltsev, A. N.; Sokolov, M. N.; Novikov, A. S.; Adonin, S. A. Iodine-Rich Supramolecular Complexes of Chlorobismuthates(III): Unusual Lone Pair Activity and Features of Halogen Bonding in Crystals. *Inorganic Chemistry* **2024**, *63* (38), 17755-17761. DOI: 10.1021/acs.inorgchem.4c02593.
(9) *Spectragryph - optical spectroscopy software*; https://www.effemm2.de/spectragryph/index.html (accessed 01/12/2024).
(10) Toby, B. H.; Von Dreele, R. B. GSAS-II: the genesis of a modern open-source all purpose crystallography software package. *Journal of Applied Crystallography* **2013**, *46* (2), 544-549. DOI: doi:10.1107/S0021889813003531.
(11) Dolomanov, O. V.; Bourhis, L. J.; Gildea, R. J.; Howard, J. A. K.; Puschmann, H. OLEX2: A complete structure solution, refinement and analysis program *J. Appl. Cryst.* **2009**, *42* (2), 339-341.
(12) Sheldrick, G. M. Crystal structure refinement with SHELXL. *Acta Crystallographica Section C Structural Chemistry* **2015**, *71* (1), 3-8.
(13) Biswas, C.; Chattopadhyay, S.; Drew, M. G. B.; Ghosh, A. Synthesis, crystal structure and hydrolysis of a dinuclear copper(II) complex constructed by N2O donor Schiff base and 4,4′-bipyridine: Discrete supra-molecular ensembles vs. oligomers. *Polyhedron* **2007**, *26* (15), 4411-4418. DOI: 10.1016/j.poly.2007.05.034.
(14) Rozhkov, A. V.; Ananyev, I. V.; Petrov, A. A.; Galmés, B.; Frontera, A.; Bokach, N. A.; Kukushkin, V. Y. Ligand Steric Hindrances Switch Bridging (μ2-I)···O,O to Two-Center I···O Halogen-Bonding Mode in the Assembly of Diketonate Copper(II) Species. *Crystal Growth & Design* **2021**, *21* (7), 4073-4082. DOI: 10.1021/acs.cgd.1c00373.
(15) Blasi, D.; Nicolai, V.; Gomila, R. M.; Mercandelli, P.; Frontera, A.; Carlucci, L. Unprecedented {dz(2)-Cu(II)O4}cdots, three dots, centeredpi-hole interactions: the case of a cocrystal of a Cu(II) bis-beta-diketonate complex with 1,4-diiodotetrafluoro-benzene. *Chem Commun (Camb)* **2022**, *58* (68), 9524-9527. DOI: 10.1039/d2cc03457c.
(16) Scheiner, S. Understanding noncovalent bonds and their controlling forces. *J Chem Phys* **2020**, *153* (14), 140901. DOI: 10.1063/5.0026168 From NLM PubMed-not-MEDLINE.
(17) Makarenko, A. M.; Trubin, S. V.; Zherikova, K. V. Breaking through the Thermodynamics "Wilds" of Metal–Organic Chemical Vapor Deposition Precursors: Metal tris-Acetylacetonates. In *Coatings*, 2023; Vol. 13.

(18) Ribeiro da Silva, M. A. V.; Monte, M. J. S.; Huinink, J. Vapour pressures and standard molar enthalpies of sublimation of seven crystalline copper(II) β-diketonates. The mean molar (Cu-O) bond-dissociation enthalpies. *The Journal of Chemical Thermodynamics* **1995**, *27* (2), 175-190. DOI: https://doi.org/10.1006/jcht.1995.0014.
(19) Torubaev, Y. V.; Skabitsky, I. V. Overlooked Solid State Structure of 1,3-I2C6F4—The Meta-Member of an Iconic Halogen Bond Donors Trio. *Crystals* **2023**, *13* (11). DOI: 10.3390/cryst13111555.
(20) De Santis, A.; Forni, A.; Liantonio, R.; Metrangolo, P.; Pilati, T.; Resnati, G. N.Br halogen bonding: one-dimensional infinite chains through the self-assembly of dibromotetrafluorobenzenes with dipyridyl derivatives. *Chemistry* **2003**, *9* (16), 3974-3983. DOI: 10.1002/chem.200204655.
(21) Walsh, R. B.; Padgett, C. W.; Metrangolo, P.; Resnati, G.; Hanks, T. W.; Pennington, W. T. Crystal Engineering through Halogen Bonding: Complexes of Nitrogen Heterocycles with Organic Iodides. *Crystal Growth & Design* **2001**, *1* (2), 165-175. DOI: 10.1021/cg005540m.
(22) Wang, R.; Hartnick, D.; Englert, U. Short is strong: experimental electron density in a very short N···I halogen bond. *Zeitschrift für Kristallographie - Crystalline Materials* **2018**, *233* (9-10), 733-744. DOI: 10.1515/zkri-2018-2069.
(23) Szell, P. M. J.; Grebert, L.; Bryce, D. L. Rapid Identification of Halogen Bonds in Co-Crystalline Powders via (127) I Nuclear Quadrupole Resonance Spectroscopy. *Angew Chem Int Ed Engl* **2019**, *58* (38), 13479-13485. DOI: 10.1002/anie.201905788 From NLM PubMed-not-MEDLINE.
(24) Quentin, J.; MacGillivray, L. R. Halogen versus Hydrogen Bonding in Binary Cocrystals: Novel Conformation a Coformer with [2+2] Photoreactivity of Criss-Crossed C=C Bonds. *ChemPhysChem* **2020**, *21* (2), 154-163. DOI: https://doi.org/10.1002/cphc.201900961 (acccessed 2024/07/30).
(25) Quentin, J.; MacGillivray, L. R. Hydrogen- and Halogen-Bonded Binary Cocrystals with Ditopic Components: Systematic Structural and Photoreactivity Properties That Provide Access to a Completed Series of Symmetrical Cyclobutanes. *Crystal Growth & Design* **2020**, *20* (11), 7501-7515. DOI: 10.1021/acs.cgd.0c01143.
